# Music reward sensitivity is associated with greater information transfer capacity within dorsal and motor white matter networks in musicians

**DOI:** 10.1007/s00429-024-02836-x

**Published:** 2024-07-25

**Authors:** Tomas E. Matthews, Massimo Lumaca, Maria A. G. Witek, Virginia B. Penhune, Peter Vuust

**Affiliations:** 1 Center for Music in the Brain, Department of Clinical Medicine, Aarhus University Hospital, Nørrebrogade 44, Building 1A, Aarhus C, 8000 Denmark; 2 Department of Music School of Languages, Art History and Music, University of Birmingham, Cultures, Birmingham, B15 2TT UK; 3 Department of Psychology, Concordia University, 7141 Sherbrooke St W, Montreal, QC H4B 1R6 Canada; 4 Royal Academy of Music, Skovgaardsgade 2C, Aarhus C, DK-8000 Denmark

**Keywords:** Musical pleasure, White matter structure, Fixel-based analysis, Musical training

## Abstract

There are pronounced differences in the degree to which individuals experience music-induced pleasure which are linked to variations in structural connectivity between auditory and reward areas. However, previous studies exploring the link between white matter structure and music reward sensitivity (MRS) have relied on standard diffusion tensor imaging methods, which present challenges in terms of anatomical accuracy and interpretability. Further, the link between MRS and connectivity in regions outside of auditory-reward networks, as well as the role of musical training, have yet to be investigated. Therefore, we investigated the relation between MRS and structural connectivity in a large number of directly segmented and anatomically verified white matter tracts in musicians (*n* = 24) and non-musicians (*n* = 23) using state-of-the-art tract reconstruction and fixel-based analysis. Using a manual tract-of-interest approach, we additionally tested MRS-white matter associations in auditory-reward networks seen in previous studies. Within the musician group, there was a significant positive relation between MRS and fiber density and cross section in the right middle longitudinal fascicle connecting auditory and inferior parietal cortices. There were also positive relations between MRS and fiber-bundle cross-section in tracts connecting the left thalamus to the ventral precentral gyrus and connecting the right thalamus to the right supplementary motor area, however, these did not survive FDR correction. These results suggest that, within musicians, dorsal auditory and motor networks are crucial to MRS, possibly via their roles in top-down predictive processing and auditory-motor transformations.

**Supplementary Information:**

The online version contains supplementary material available at 10.1007/s00429-024-02836-x.

There are pronounced interindividual differences in the degree to which humans derive pleasure from their experiences, even in non-clinical populations (Harvey et al. 2007). This is particularly the case for music (Mas-Herrero et al. 2013, 2014). Interindividual differences in hedonic responses to music have been linked to differences in brain activity, connectivity, and structure, particularly within auditory-reward networks (Hernández et al. 2019; Loui et al. 2017; Martínez-Molina et al. 2016, 2019; Quinci et al. 2022; Sachs et al. 2016). This suggest that the degree to which individuals gain pleasure from music, known as music reward sensitivity (MRS), relies on the transfer of music perceptual information from auditory cortices to areas that process the reward value of this information. However, other cognitive processes, such as those involved in prediction and anticipation, have been strongly linked to musical pleasure, and are associated with regions outside of auditory-reward networks (Mas-Herrero et al. 2018, 2021; Salimpoor et al. 2011, 2013). Further, musical training is linked to higher MRS (Hernández et al. 2019), differences in brain activity and structure, and cognitive and perceptual processes that could influence MRS (Brown et al. 2015; Criscuolo et al. 2022). However, the influence of musical training on the structural correlates of MRS has yet to be investigated. Previous studies relating white matter microstructure to MRS have relied on diffusion tensor imaging-based analysis methods which are limited in both their anatomical accuracy and interpretability in terms of information transfer capacity (Jones et al. 2013). Here, we used state of the art, fixel-based analysis to investigate the association between MRS and the structure of a large number of anatomically verified tracts in both musicians and non-musicians.

MRS, measured using the Barcelona Music Reward Questionnaire (BMRQ), describes a relatively stable trait that reflects individuals’ general engagement (or lack thereof) with music (Mas-Herrero et al. 2013). In terms of white matter structural connectivity, BMRQ scores correlate with greater tract volume and lower axial diffusivity in tracts connecting auditory cortices (posterior superior temporal gyrus; pSTG) with the anterior insula, nucleus accumbens (NAcc), and medial prefrontal cortex (including the orbitofrontal cortex), as well as between the medial prefrontal cortex and NAcc (Loui et al. 2017; Martínez-Molina et al. 2019). In addition, Sachs et al. (2016) found that the frequency at which participants experience pleasurable ‘chills’ in response to self-selected music was positively correlated with the volume of a tract connecting pSTG, insula, and medial prefrontal cortex in the right hemisphere. Together these studies suggest that information transfer between auditory and reward regions, such as the ventral striatum and medial prefrontal cortex, particularly in the right hemisphere, is crucial to MRS. This is broadly supported by functional neuroimaging studies that show a link between activity in these same regions and felt pleasure during music listening (see Mas-Herrero et al. 2021 for a meta-analysis).

Given the multi-dimensionality and subjectivity of the enjoyment of music, processes and brain networks beyond those directly involved in auditory perception and the computation of reward are also likely to play a role in MRS. This is supported by work showing that patients with acquired musical anhedonia due to brain damage exhibit lesions in a distributed network including parietal and somatosensory cortices, posterior cingulate gyrus, and prefrontal regions (Belfi et al. 2017; Johnsen et al. 2009; Mazzoni et al. 1993; Satoh et al. 2011). Further, functional neuroimaging studies show that, in addition to auditory and reward networks, musical pleasure is associated with activity in motor, dorsal, and frontoparietal networks (Ara and Marco-Pallarés 2020, 2021; Blood and Zatorre 2001; Chabin et al. 2020; Koelsch et al. 2021; Kornysheva et al. 2010; Mas-Herrero et al. 2018, 2021; Matthews et al. 2020; Singer et al. 2016). One possible explanation is based on theories suggesting an important role of predictive processes in musical pleasure (Huron 2006; Koelsch et al. 2019; Meyer 1956; Pearce and Wiggins 2012; Salimpoor et al. 2015; Vuust et al. 2022; Zald and Zatorre 2011). Recent meta-analyses show that the patterns of activations associated with producing, imagining, and listening to music overlap strongly with a modality-general prediction network (Pando-Naude et al. 2021; Siman-Tov et al. 2019), including cortical-basal ganglia-thalamic, frontoparietal, and dorsal auditory networks. These networks are implicated in the temporal predictions and auditory-motor transformations necessary to perceive and synchronize to musical rhythms (Alluri et al. 2017; Cannon and Patel 2020; Chen et al. 2008; Grahn and Brett 2007; Grahn and Rowe 2009; Kasdan et al. 2022; Kung et al. 2013; Schubotz 2007; Schubotz et al. 2000; Thaut et al. 2014; Toiviainen et al. 2019) and the generation and violation of melodic and harmonic predictions (Bianco et al. 2016; Cheung et al. 2019; Lappe et al. 2013, 2016; Li et al. 2021; Seger et al. 2013). Further, these networks are thought to support the memory and attentional processes necessary to generate and update internal representations of music and to parse its syntactic structure (Gelding et al. 2019; Herholz et al. 2012; Jiang et al. 2023; Zhang et al. 2017). We hypothesize that differences in the tendency or degree to which individuals engage in these predictive, attentional, memory, and auditory-motor processes during music listening influences their MRS, and that this differential engagement is reflected in white matter structure.

Musical training is likely to moderate the relation between MRS and the engagement of the processes described above. Through extensive practice and/or predisposition, musicians show stronger auditory-motor associations and refined predictive and perceptual processes compared to non-musicians (Alluri et al. 2017; Hansen et al. 2022; Jiang et al. 2023; Jongsma et al. 2004, 2005; Luo et al. 2012; Palmer and Krumhansl 1990; Pesnot Lerousseau and Schön 2021; Rigoulot et al. 2015; Vuust et al. 2012). Musicians also show greater activation and greater functional connectivity among auditory, motor, parietal, and prefrontal regions while listening to music (Alluri et al. 2017; Criscuolo et al. 2022; Grahn and Rowe 2009; Matthews et al. 2020; Singer et al. 2016). In terms of structural differences, musical training is associated with changes in gray and white matter (Baer et al. 2015; Bermudez and Zatorre 2005; Cheng et al. 2022; Vaquero et al. 2015) including altered white matter microstructure within motor tracts and interhemispheric auditory tracts (Leipold et al. 2021; Steele et al. 2013; Vollmann et al. 2014). Musical training is also associated with altered affective responses to music, including higher MRS overall (Hernández et al. 2019) and greater liking of musical stimuli (Matthews et al. 2019). Musicians also show a stronger pleasurable urge to move to more complex musical stimuli (Matthews et al. 2019, 2022), a response that is linked to predictive processes and activity within motor and reward networks (Koelsch et al. 2019; Matthews et al. 2020; Stupacher et al. 2022; Vuust et al. 2022; Vuust and Witek 2014). However, it is unclear if musical training plays a role in the link between MRS and white matter microstructure.

Diffusion tensor imaging (DTI)-based measures have been crucial in illuminating individual differences in white matter structure, including those related to MRS (Loui et al. 2017; Martínez-Molina et al. 2019; Sachs et al. 2016). However, the link between these measures and the capacity of white matter tracts to relay information is somewhat tenuous as differences in these measures can be driven by several factors including myelination, axon density, and the complexity of fiber geometries (Jones et al. 2013). One of the primary limitations of DTI-based measures is their inability to resolve crossing fibers within a voxel (Jones 2010), which, given that up to 90% of voxels contain crossing fibers (Jeurissen et al. 2013), reduces the biological specificity of associated white matter metrics and has detrimental effects on processing techniques such as streamlined tractography (Farquharson et al. 2013). For this reason, new approaches have been developed such as fixel-based analysis, which uses a more advanced diffusion model, constrained spherical deconvolution, that can address the issue of crossing fibers more effectively (Raffelt et al. 2015; Raffelt, Tournier, Smith, Vaughan, Raffelt et al. 2017a, b). In addition, fixel-based analysis provides structural measures specific to axon fiber bundles within voxels (hence fixels) and is carried out only on fiber bundles that have been determined to be in the expected orientation. Fixel-based analysis focuses on two indices: fiber density and fiber cross-section, along with the combination of the two. Fiber density provides a microstructural measure linked to the number and diameter of axons within a fixel while fiber cross-section provides a macrostructural measure of the cross-sectional area of the fiber bundle. Combining these measures provides a comprehensive index of intra-axonal volume which can be interpreted in terms of information transfer capacity (Dhollander et al. 2021; Raffelt et al. 2017a, b).

In the current study, we used fixel-based analysis of white matter microstructure to investigate the relation between MRS and structural connectivity, in directly segmented and anatomically verified white matter tracts. A hypothesis-driven set of tracts included those within auditory-reward networks as well as cortico-basal ganglia-thalamo-cortical and dorsal/frontoparietal networks associated with music-relevant predictive, attentional, memory, and auditory-motor processes. An additional exploratory set of tracts included those within networks related to predictive and affective processes, including thalamo-cortical and cerebellar tracts (Blood and Zatorre 2001; Brown et al. 2004; Gatti et al. 2021; Kasdan et al. 2022; Menon and Levitin 2005). These relations were investigated in musicians and non-musicians separately as well as in the entire sample with both groups combined. Based on evidence of the important role of auditory-reward networks in MRS, which may not be fully captured by the above tracts (Loui et al. 2017; Martínez-Molina et al. 2019; Sachs et al. 2016), we also carried out ROI-based analysis on tracts connecting orbitofrontal cortex with auditory cortex and NAcc. Given the significant methodological differences from those previous studies, here we attempt a conceptual rather than direct replication. As the combined cross-section and density measure can be interpreted in terms of information transfer capacity, we hypothesized a positive relation between this measure and MRS in all tracts tested.

## Methods

### Participants

Fifty-seven right-handed young adults (musicians and non-musicians) were recruited for a separate functional magnetic resonance imaging study (Matthews et al. 2020). Of these, 54 participants underwent diffusion magnetic resonance imaging (dMRI). Following initial data quality checks, a sample of 24 musicians and 23 non-musicians were included in the final analysis. Table 1 shows demographic and musical training data for the final sample. All non-musicians had a year or less of formal musical training and were not regularly practicing an instrument at the time of data collection. All musicians had been playing an instrument or singing regularly for at least 8 years and were practicing regularly when data was collected. The musicians were primarily instrumentalists (five were vocalists). Eight musicians reported playing classical music as their primary genre and the rest reported a variety of genres, including jazz, pop, and funk. All participants reported no hearing problems, nor any neurological or psychiatric disorders. Informed consent was obtained, and the study was approved by the Central Denmark Region Committees on Health Research Ethics. Participants received 200 DKK remuneration.

**Table 1:** Participant demographic data

	Non-Musicians	Musicians
N (male/female)	23 (12/11)	24 (13/11)
Age (SD; range)	23.04 (2.50; 20–27)	23.38 (2.83; 18–31)
BMRQ (SD; range)	67.65 (13.89; 38–97)	82.79 (9.08; 64–98)
Years of musical training (SD; range)	0.17 (0.32; 0–1)	11.27 (3.00; 6–18)
Years playing music (SD; range)		14.42 (3.94; 8–25)
Hours of music practice per week (SD; range)		10.63 (10.14; 1–35)
Age of start (SD; range)		8.35 (3.13; 2–16)

*Note* SD, standard deviation; BMRQ, Barcelona Music Reward Questionnaire

**Fig. 1:**
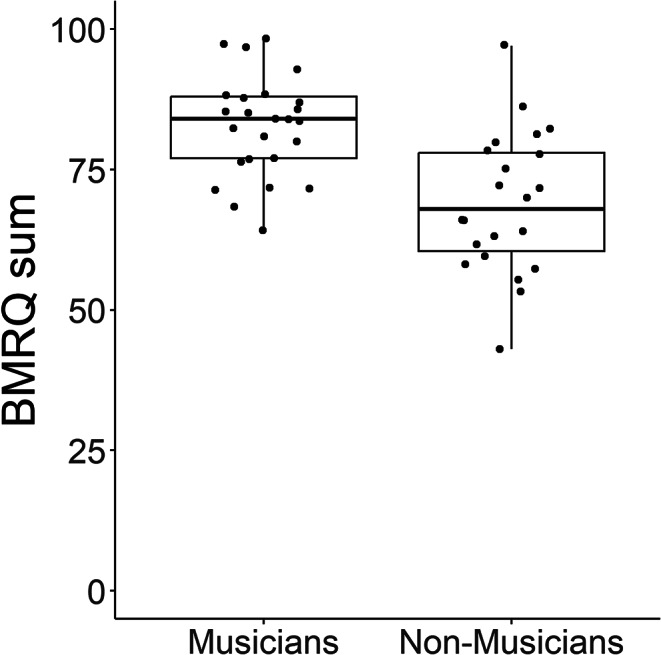
Boxplots of BMRQ scores for musicians and non-musicians. Center line, median; box limits, upper and lower quartiles; whiskers, 1.5x interquartile range

All participants completed the Barcelona Music Reward Questionnaire (BMRQ; Mas-Herrero et al. 2013). The BMRQ is a validated questionnaire for measuring MRS that consists of 20 items covering five aspects of music reward, including emotional evocation, sensory-motor, musical seeking, mood regulation, and social reward. For each item, participants indicate their level of agreement on a scale of 1 to 5, where 1 corresponds to ‘fully disagree’ and 5 corresponds to ‘fully agree’. After reversing the scores of the two reverse items, scores were summed to get an overall measure of MRS. Figure 1 shows BMRQ scores for both groups. A Welch’s two sample t-test showed that musicians had a significantly higher mean score compared to non-musicians (*t* = 4.40, *p* < .001).

### dMRI acquisition

The acquisition of diffusion weighted images was performed with a 3T MRI Siemens TIM Trio scanner. We acquired single-shell diffusion images that included: 62 diffusion directions at b = 1500 s/mm^2^; 9 directions at b = 5 s/mm^2^ (flip angle = 90°, TR/TE = 4200/91 ms, voxel size = 2 × 2 × 2 mm^3^, matrix size = 96 × 96, number of slices = 66). The phase-encoding direction of those data was anterior–posterior. In addition, a reversed b = 5 s/mm^2^ image was acquired with opposite phase-encoding direction (i.e., posterior-anterior) for the purpose of EPI distortion correction (Andersson et al. 2003; Holland et al. 2010).

### dMRI preprocessing and fixel metrics

Diffusion data was preprocessed using the publicly available fixel-based analysis pipeline (https://www.mrtrix.org*)* with the MRtrix3 software package (Version 3.0_RC3; Tournier et al. 2019). MRtrix3 scripts also interfaced with external software packages such as FSL (Jenkinson et al. 2012) and ANTS N4 (Avants et al. 2014).

In brief, dMRI data were denoised (Veraart et al. 2016), removed of Gibbs ringing artifacts (Kellner et al. 2016) corrected for eddy, motion, and susceptibility EPI-induced distortions (Andersson et al. 2003; Andersson and Sotiropoulos 2016), and for spatial intensity inhomogeneity (Tustison et al. 2010). A global intensity normalisation was performed on preprocessed images across subjects by dividing all volumes by the median b = 0 s/mm2 intensity within a white-matter mask (Raffelt et al. 2017a, b). Next, an unsupervised approach (“dhollander”) was used to obtain three-tissue response functions representing single-fiber white matter, gray matter, and cerebral spinal fluid (Dhollander et al. 2016). Tissue response functions for white matter and cerebrospinal fluid were then averaged across participants. Subsequently, dMRI data were upsampled to an isotropic voxel size of 1.3 mm^3^ using the (default) cubic interpolation method to improve the downstream generation of a population template. Upsampled white matter masks were then estimated. Multi-tissue constrained spherical deconvolution (Tournier et al. 2007) was used to compute fiber orientation distributions (FODs) using the two group tissue responses. A study-specific FOD population template was generated through linear and non-linear registrations of 40 subjects’ white matter FODs (randomly sampling 20 musicians and 20 non-musicians; Raffelt et al. 2011).

We then followed the pipeline outlined in Fuelscher et al. (2021) to produce fixel-based metrics in the MNI space. Specifically, we first co-registered the study-specific population template to MNI space using fractional anisotropy based affine registration in FSL. Then, we registered the participants’ FOD images to the new template space and segmented them into discrete fixels (Smith et al. 2013). Fiber density (FD), fiber cross section (FC), and fiber density and cross section (FDC) were calculated in every white-matter fixel for each subject (Tournier et al. 2019). Fixel directions were then re-oriented and a fixel-fixel correspondence was obtained to the template image.

### Automatic definition of tracts-of-interest using TractSeg

TractSeg (Wasserthal et al. 2018) was used to generate streamlines and to reconstruct 72 white-matter tracts in the MNI template space. Based on evidence implicating cortico-striatal networks in the predictive processes underlying music-induced reward (Cheung et al. 2019; Gold et al. 2019, 2023; Martínez-Molina et al. 2016, 2019; Mas-Herrero et al. 2018; Salimpoor et al. 2011, 2013; Shany et al. 2019), we selected seven tracts of primary interest: striato-fronto-orbital, striato-prefrontal, striato-premotor, striato-precentral, striato-parietal, inferior occipito-frontal fascicle, and uncinate fascicle. In addition, due to the importance of the dorsal auditory stream in music perception and production (Warren et al. 2005; Zatorre et al. 2007), we hypothesized the involvement of the middle longitudinal fascicle, superior longitudinal fascicle I and II. In addition to these tracts, we included 11 relevant tracts for exploratory analysis: superior longitudinal fascicle III, arcuate fascicle, inferior cerebellar peduncle, superior cerebellar peduncle, thalamo-parietal, thalamo-postcentral, thalamo-precentral, thalamo-premotor, thalamo-prefrontal, anterior thalamic radiation, and superior thalamic radiation. These tracts were selected based on their involvement in auditory-motor mapping (arcuate fascicle, superior longitudinal fascicle III) and motor-predictive processes (thalamic and cerebellar tracts). Before each analysis, fixel-masks were cropped to only include fixels that belonged to the tracts of interest. All tracts were visually checked before performing fixel-based analysis and are shown in Figure S1.

### Manual definition of tracts of interest

Based on previous studies (Loui et al. 2017; Martínez-Molina et al. 2019; Sachs et al. 2016), we investigated whether fixel-based metrics were also related to neuroanatomical bundles connecting auditory and reward regions. We used a manual procedure to reconstruct tracts connecting pSTG to orbital frontal cortex (OFC), and OFC to NAcc. A whole-brain tractogram with 10 million streamlines was generated with dynamic seeding using the default second-order over Fibre Orientation Distribution (iFOD2) tractography algorithm (length of streamlines, 10–250 mm; maximum angle, 22.5 degrees, step size 0.5 mm). We applied the spherical-deconvolution informed filtering of tractograms (SIFT) algorithm (Smith et al. 2013) to filter and refine the tractogram, reducing it to 1 million streamlines. This method ensures that the streamline density aligns well with the underlying fiber density estimated in the step of spherical deconvolution. We then reconstructed the pathways of interest by selecting the SIFT-based streamlines connecting OFC to NAcc and pSTG to OFC. Spherical ROIs (12 mm in diameter) were centered on the following MNI coordinates: left NAcc (*x y z*: -8.03, 8.74, -5.81), right NAcc (9.46, 8.73, -5.26), left OFC (-28.67, 23.98, -13.92), right OFC (29.06, 23.15, -13.79), left pSTG (-61.32, -27.37, 5.66), and right pSTG (60.88, -22.95, 4.31). These coordinates were extracted from the centroids of the anatomical masks corresponding to these regions and obtained from the Harvard-Oxford cortical and subcortical atlases (https://fsl.fmrib.ox.ac.uk/). A fixel-based mask was obtained from the extracted streamlines and used for the statistical analyses of fixel-based metrics. Averages of these metrics were calculated across all streamlines.

### Statistical analysis

We used a General Linear Model, separately for musicians and non-musicians, to relate the three fixel-based metrics to individual BMRQ scores, while including age, gender, and relative head motion as nuisance regressors (Bastiani et al. 2019; Lumaca et al. 2021). We also used the same approach in the entire sample including both groups together. Connectivity-based fixel enhancement with default parameters and non-parametric permutation-based hypothesis testing (*n* = 5000) were performed for each fixel-based metric (Nichols et al., 2002; Raffelt et al. 2015; Winkler et al., 2014). At the end of this process, each fixel from the tract of interest had an assigned family-wise error corrected (FWE) *p*-value. A separate analysis was conducted for each tract. Given that we hypothesized a positive relation between MRS and fixel-based metrics, only one-tailed tests were performed. To address the multiple comparison problem (number of tests = 21), we performed FDR correction (*q* < 0.05) on the FWE-corrected p-values of the peak fixels within each tract (Benjamini & Hochberg, 1995). We considered significant only the tracts whose FWE-corrected p-values in the peak fixels survived the FDR correction.

To visualize the relation between MRS and fixel-based metrics, controlling for age and gender, partial correlations were calculated using the per-participant mean of the fixel-based metric. Corresponding scatterplots were generated by calculating the residuals of two linear regression models, one with age and gender predicting BMRQ score and the other with age and gender predicting the fixel-based metric.

For the manually defined tracts of interest, the respective metrics were averaged over all streamlines within the extracted tracts, resulting in one metric-specific value per tract, per participant. These values were then submitted to regression analyses, with one regression model per tract, BMRQ score as the dependent variable, and fixel-based metrics (FC, FD, and FDC) as predictors. It should be noted that tractography resulted in very few streamlines between STG and OFC in both hemispheres. However, analyses were carried out on these tracts to match the tracts analyzed by Martínez-Molina et al. (2019). Further, Martínez-Molina et al. (2019) showed that the STG-OFC voxels showing significant relation to BMRQ overlapped strongly with IFO, a tract that was also tested here in the main analysis. Therefore, these tract-of-interest analyses are considered supplementary to the main analyses.

## Results

**Fig. 2:**
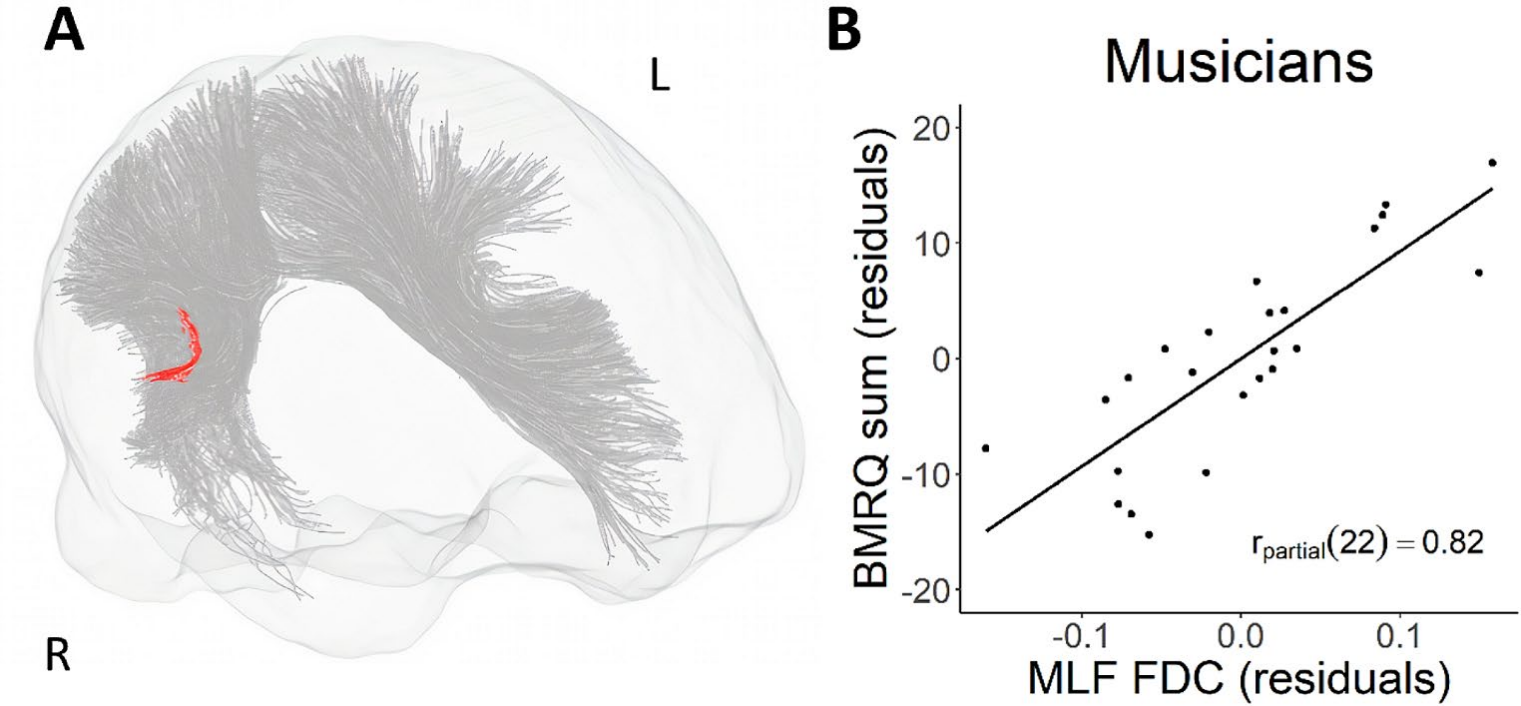
**A**) Fixels within the MLF for which FDC showed a significant positive relation with BMRQ superimposed on the MLF and AF tracts. **B**) A scatterplot shows partial correlation between the per-participant mean values of FDC within MLF and BMRQ, controlling for age and gender. MLF, middle longitudinal fascicle; FDC, fiber density and cross section; AF, arcuate fasciculus; BMRQ, Barcelona Music Reward Questionnaire

**Fig. 3:**
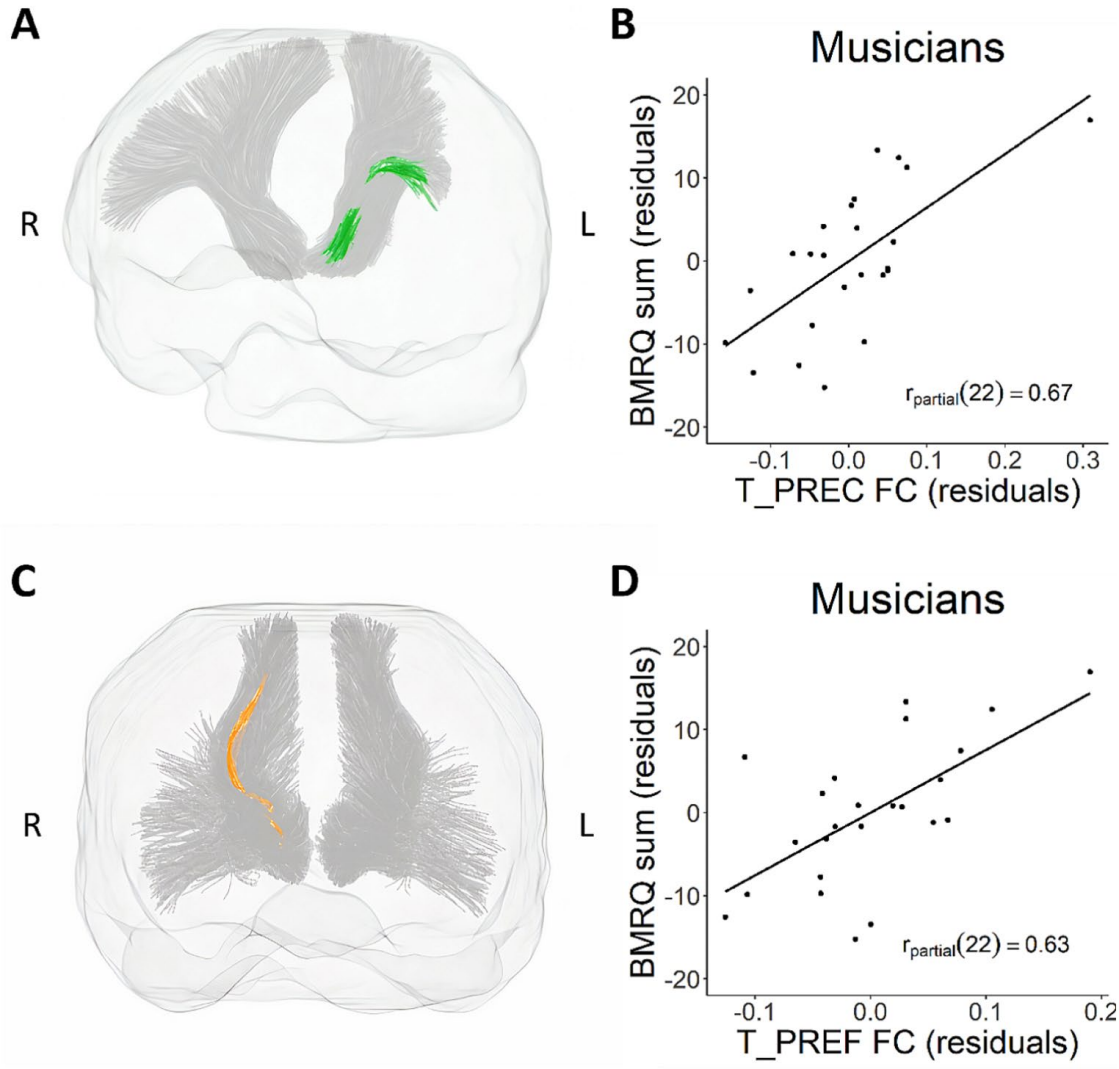
**A**) and **C**) Fixels within thalamo-precentral and thalamo-prefrontal tracts for which FC showed a significant positive relation with BMRQ, superimposed on their respective tracts. **B**) and **D**) Scatterplots showing partial correlations between the per-participant mean values of FC within thalamo-precentral and thalamo-prefrontal, and BMRQ, controlling for age and gender. Note that these results did not survive the tract-level FDR correction for multiple comparisons. Removing the apparent outlier in **B**) results in a partial correlation of r_partial_(21) = 0.60. T_PREC, thalamo-precentral tract; T_PREF, thalamo-prefrontal tract; FC, fiber cross-section

Musicians showed a significant positive relation between BMRQ scores and FDC in the right middle longitudinal fascicle (MLF; see Table 2; Fig. 2). Musicians also showed a significant positive relation between BMRQ scores and FC in a large number of fixels in the left thalamo-precentral (Fig. 3A) and right thalamo-prefrontal (Fig. 3B), however these results did not survive tract-level FDR correction for multiple comparisons. In addition, musicians showed a positive relation between BMRQ score and fixel-based metrics in a smaller number of fixels in left striato-precentral (FDC, FC), left thalamo-precentral (FDC), left arcuate fascicle (FD), the superior thalamic radiation (FC), right thalamo-premotor (FC), and left thalamo-postcentral (FC; Table 2), which also did not survive FDR correction at the tract level. The non-musician group showed no significant association between BMRQ and fixel-based metrics in any of the tracts tested. There were no significant associations between BMRQ scores and fixel-based metrics in any of the tracts when testing in both groups together. Comparing musicians and non-musicians showed no significant differences.

**Table 2:** Tracts showing significant fixels (FWE corrected) in musicians

Fixel metric	Tract name	Number of significant fixels (*P*_FWE_<0.05)	*P*_FWE_peak fixel	F-statistics (averaged across significant fixels)
*FDC*	*middle longitudinal fascicle (R)	91	0.001	3.94
	striato-precentral (L)	10	0.032	3.09
	thalamo-precentral (L)	6	0.047	2.82
*FD*	arcuate fascicle (L)	6	0.019	3.95
*FC*	thalamo-precentral (L)	437	0.008	3.02
	thalamo-prefrontal (R)	155	0.023	3.05
	superior thalamic radiation (B)	14	0.038	2.42
	thalamo-premotor (R)	12	0.043	2.61
	striato-precentral (L)	5	0.032	3.78
	thalamo-postcentral (L)	5	0.043	2.95

*Note* * tract whose P_FWE_ survived tract level FDR correction

### Effects of musical training

A correlation analysis within the musician group did not reveal any strong or significant correlations between musical training variables and BMRQ scores. The highest correlation was between BMRQ and years playing music (*r*(22) = 0.22, *p* = .30), while years of formal music training (*r*(22) = 0.06, *p* = .76), age of start (*r*(22) = 0.12, *p* = .57), and hours practicing per week (*r*(22) = 0.14, *p* = .52) showed weaker correlations with BMRQ. This suggests that the relation between MRS and fixel-based metrics was not confounded with the relation between MRS and musical training.

However, since only musicians showed a significant relation between MRS and fixel-based metrics, we conducted additional analyses to investigate the relation between musical training measures and fixel-based metrics. We assessed the relation between years of formal music training and fixel-based metrics in musicians and non-musicians (for completeness) separately, as well as with both groups together, with music training (demeaned) as the explanatory variable, and age, gender, and relative head motion as covariates. We also tested the relation between fixel-based metrics and years playing music, age of start, and hours of weekly practice in musicians only, in three separate analyses, with age, gender, and relative head motion as covariates. As with the previous analyses, we implemented FWE correction for multiple within-tract comparisons and FDR correction over the 21 tracts of interest, for each musical training variable separately.

For years of formal music training, no associations between this measure and fixel-based metrics survived FDR correction in any of the tracts tested, when analyzing either group separately or both groups together. In the musicians-only analyses, only the relation between years playing music and FC in the striato-premotor tract survived FDR correction (*F* = 3.89, *p*_*FDR*_ = 0.038, n_Fixels_ = 801; see Fig S3). Notably these fixels did not overlap with those showing a significant relation with BMRQ scores.

### Manually defined tracts of interest

Additional analyses were carried out on manually defined tracts of interest based on previous studies showing significant relations between BMRQ and DTI-based metrics of white matter structure in auditory-reward tracts (Loui et al. 2017; Martínez-Molina et al. 2019; Sachs et al. 2016). These tracts connect ROIs, including pSTG and OFC, and NAcc and OFC.

In the tract connecting left NAcc and OFC, an initial model with all predictors and interactions showed a significant main effect of FDC (*F*(1, 39) = 5.16, *p* = .029) and a significant group by FD interaction (*F*(1, 39) = 7.38, *p* = .010). However, in a follow-up model with only these effects, along with the main effect of group, the main effect of FDC was no longer significant (*F*(1, 42) = 1.26, *p* = .269). Further, this model showed only a near-significant group by FD interaction (*b* = 508.32, *t* = 1.854, *p* = .071), driven by musicians showing a negative relation and non-musicians showing a positive relation. This divergence between initial and final models suggests that the results of the initial model were relatively unstable and likely influenced by colinearity between the predictor variables as FC and FDC are highly correlated (*r* = .91). Therefore, FC was likely influencing the effects of FD and FDC in the initial model despite not being itself involved in any significant effects. There was also a main effect of FDC in the tract connecting right STG and OFC. However, a model with only the main effect of group and FDC showed no significant relation between FDC and BMRQ, again likely due to the multiplicative effects of colinearity between FDC and FC. To test for group differences, we carried out a mixed-design ANOVA with group as the between-group factor, tract measure (e.g., STG-OFC FDC) as the within-group factor, and FDC, FC, and FD values as the dependent variable. The group by tract measure interaction was not significant (*F*(2.68, 120.42) = 0.148, *p* = .914), indicating that there were no significant between-group differences in the fixel-based metric in these tracts.

## Discussion

In this study, we investigated the hypothesis that MRS relies on dorsal and motor networks by applying fixel-based analysis on a large number of anatomically defined white matter tracts in musicians and nonmusicians. Musicians showed a positive relation between MRS and fiber density and cross-section (FDC), a proxy of information transfer capacity (Raffelt et al. 2017a, b), in a subsection of the right middle longitudinal fasciculus (MLF). Extending the tracts that pass through significant fixels suggested terminations in the pSTG (BA 22), likely the planum temporale within the auditory cortex, and the ventral supramarginal gyrus (BA 40), which overlaps with the temporoparietal junction and inferior parietal lobule (Figure S2A). Musicians also showed a positive relation between MRS and FC, which is a macrostructural metric of fiber bundle area. This relation was observed in a large number of fixels within white matter tracts from the left thalamus (BA 50) with apparent terminations in the left ventral precentral gyrus/inferior frontal gyrus (BA 44; Figure S2B), and the right thalamus (BA 50) with apparent terminations in the right supplementary motor area (BA 6; Figure S2C). However, these results did not survive tract-level FDR correction for multiple comparisons. Follow-up analyses confirmed that these relations were unlikely to be driven by musical training. Further supplementary analyses were carried out on manually defined tracts of interest connecting auditory and reward ROIs. There was a non-significant trend indicating that musicians show negative relation and non-musicians show a positive relation between FD and MRS in a tract connecting the left NAcc and OFC.

Together, these results extend previous work on the neuroanatomical correlates of MRS and support our hypothesis that MRS is linked to information transfer within dorsal auditory and motor cortico-basal ganglia-thalamo-cortical (mCBGT) networks. That these relations were confined to the musician group suggests that only those with extensive musical training show systematic covariance between MRS and the structural properties of these tracts. Information transfer within dorsal and motor networks may contribute to the auditory-motor and predictive processes necessary to extract and exploit the hierarchical structure of music, which in turn drive pleasurable responses.

The MLF is considered part of the dorsal auditory stream (Wang et al. 2013), a large, multi-tract network connecting posterior auditory cortices to premotor and prefrontal cortices, via the inferior parietal regions (Hickok and Poeppel 2007, 2016; Merchant and Honing 2014; Rauschecker and Scott 2009). Broadly, the dorsal auditory stream is implicated in the integration of auditory and other sensory information to inform motor representations of both speech and music (Rauschecker and Scott 2009; Hickok and Poeppel 2007, 2016). Involvement of the right MLF aligns with work suggesting that the right auditory cortices are specialized for spectro-temporal processing of the lower frequencies (i.e., 1–4 Hz) that are particularly prominent in music (Albouy, Morillon, & Zatorre, 2020; Zatorre and Belin 2001). The relation between MRS and FC in Thalamo-precentral and -prefrontal tracts further implicates the dorsal auditory stream as well as the mCBGT (Alexander et al. 1986; Haber 2016; Parent and Hazrati 1995; Schwartze and Kotz 2013). Together, the dorsal auditory stream and mCBGT are implicated in the sequencing and temporal prediction processes necessary for perceiving, producing, and synchronizing to music (Agosta et al. 2017; Araneda et al. 2016; Battelli et al. 2007; Cannon and Patel 2020; Chauvigné et al. 2014; Coull et al. 2013; Grahn and Brett 2007; Grahn and Rowe 2009; Karabanov et al. 2009; Kasdan et al. 2022; Merchant and Honing 2014; Pando-Naude et al. 2021; Schubotz et al. 2000; Schwartze and Kotz 2013; Wiener et al. 2010) and have been linked to the pleasurable urge to move to music (Matthews et al. 2020). Notably, regions within dorsal auditory and motor networks show functional and structural connectivity to reward regions, and activity and functional connectivity in these networks are positively associated with the experience of musical pleasure (Ara and Marco-Pallarés 2020, 2021; Blood and Zatorre 2001; Chabin et al. 2020; Koelsch et al. 2021; Kornysheva et al. 2010; Matthews et al. 2020; Rogenmoser et al. 2016; Salimpoor et al. 2013).

Accordingly, one interpretation of the current results is that MRS is related to the predictive, attentional, and memory processes necessary to track and capitalize on the temporal structure of music (Mas-Herrero et al. 2018; Salimpoor et al. 2015). This aligns with empirical and theoretical work suggesting that predictive processes are a strong driver of music induced pleasure (Huron 2006; Koelsch et al. 2019; Meyer 1956; Salimpoor et al. 2015; Vuust et al. 2022; Zald and Zatorre 2011), and that this link is enhanced in musicians (Matthews et al. 2019, 2022). This interpretation is further supported by studies linking both activation and white matter microstructure within the dorsal auditory and motor networks to predictive processes generally (Masina et al. 2022; Schiffer and Schubotz 2011; Siman-Tov et al. 2019), and musical predictive processes in particular (Di Liberto et al. 2020; Oestreich et al. 2019; Quiroga-Martinez et al. 2020; Vaquero et al. 2021; Vuust et al. 2005). This also aligns with work relating inferior parietal and inferior frontal gyrus activity with musical working memory (Albouy et al. 2017; Foster et al. 2013; Konoike et al. 2015; Kumar et al. 2016; Teki and Griffiths 2016) and familiarity (Vuong et al. 2023), as well as the link between familiarity and musical pleasure (Ara and Marco-Pallarés 2021; Freitas et al. 2018; Green et al. 2012; Groussard et al. 2010; Jagiello et al. 2019; Quinci et al. 2022).

An alternative interpretation is that these results reflect a more direct relation between auditory-motor mapping processes and MRS. This follows theories suggesting that music is understood and elicits emotion via embodied processes such as covert action simulation (Céspedes-Guevara and Dibben 2022; Juslin 2013; Leman et al. 2018). This interpretation is supported by the strong overlap between the mirror neuron system and motor and dorsal networks (Rizzolatti and Craighero 2004). Further, musicians, who have strong auditory-motor associations and higher MRS, show greater activation of these regions during music listening (Alluri et al. 2017; Chabin et al. 2020; Hernández et al. 2019; Matthews et al. 2020; Singer et al. 2016).

Our results suggest that a systematic link between MRS and variability in white matter tracts supporting predictive and auditory-motor processes is only present in those with a significant amount of musical training. This may be due to richer or more nuanced representations of music in musicians which increases the emotional and aesthetic aspects of the musical experience, and thus MRS. Indeed, music practice strengthens auditory-motor coordination and pitch acuity (Vuust et al. 2009; Zatorre et al. 2007), which are linked to activity and white matter differences in dorsal and auditory networks (Bengtsson et al. 2005; Bianco et al. 2021; Blecher et al. 2016; Halwani et al. 2011; Imfeld et al. 2009; Loui et al. 2009; de Manzano and Ullén 2018; Oechslin et al. 2010; Steele et al. 2012). However, only weak correlations between musical training variables and BMRQ scores were found in this sample of musicians. Further, no between-group differences were detected in any of the tracts tested here. There was a significant relation between years playing music and FC in a tract connecting the left striatum and ventrolateral premotor cortex in musicians, however, these fixels did not overlap with those associated with BMRQ. Together, these results indicate that the association between MRS and white matter structure found here were not confounded with musical training variables. This may suggest that the significant MRS-white matter relation seen in musicians is driven by innate structural differences that impart a predisposition for musical engagement, rather than an effect of training-related plasticity (Mosing et al. 2016; Scarr and Mccartney 1983; Tan et al. 2014). However, the current results cannot completely discount an effect of training as the populations sampled here represent two extremes in terms of musicianship such that this data was not well-suited for testing musical training as a continuous regressor. Further, several tracts that have shown changes in white matter due to musical training, including ventral tracts, the corpus callosum, and internal capsule (Bengtsson et al. 2005; Giacosa et al. 2016; Han et al. 2009; Oechslin et al. 2018; Steele et al. 2013; Vollmann et al. 2014), were not tested here. Finally, the musician group included both instrumentalists (*n* = 19) and vocalists (*n* = 5), and previous studies have shown differences in white matter structure between these groups (e.g., Halwani et al. 2011). However, it is not clear if or how these differences might relate to MRS, therefore, future work should investigate the impact of instrument-specific training on the connection between white matter structure and MRS.

Although an initial model showed a significant relation between MRS and fixel-based metrics in manually defined auditory-reward tracts, these did not survive follow-up analysis. Also, fixel-based metrics within automatically segmented tracts such as the inferior occipito-frontal fascicle, which has been shown to overlap strongly with tracts connecting STG and OFC identified via ROI-based targeted tractography (Martínez-Molina et al. 2019), did not show a significant relation with MRS. This was regardless of whether analyzing groups separately or pooling both groups together. In addition, we found significant MRS-white matter relations only in musicians, and only outside of auditory-reward networks. This contrasts with studies that showed significant relations between MRS and DTI-based metrics in these tracts (Loui et al. 2017; Martínez-Molina et al. 2019; Sachs et al. 2016), despite participants here showing a similar range in BMRQ scores and the sample sizes being larger or comparable to those studies. However, previous studies recruited mostly non-musicians and restricted their analyses to auditory-reward networks. There were also significant methodological differences from previous studies including nearly every aspect of the preprocessing and analysis pipelines, thus allowing only for a conceptual rather than direct replication in the current study. One possibility is that previous results may have reflected white matter differences not specifically related to intra-axonal volume and information transfer capacity. However, it is important to point out that the crucial role of auditory-reward networks in MRS is bolstered by work showing functional connectivity in these networks (Martínez-Molina et al. 2016) and that axial diffusivity in tracts connecting NAcc with OFC and STG with OFC mediates the relation between NAcc activation and MRS (Martínez-Molina et al. 2019). Therefore, future work should investigate the relation between fixel-based metrics with functional connectivity in dorsal and mCBGT networks, along with subjective and physiological measures of musical pleasure in participants with a large range of musical training.

Contrary to our hypothesis we did not see a link between MRS and fixel-based metrics in striato-cortical tracts. However, we did see a positive relation between MRS and FC, a macrostructural measure of fiber bundle diameter, in thalamo-premotor and thalamo-prefrontal tracts. Classic models of CBGT circuits suggest that striatal regions receive input from the cortex then, after processing within other basal ganglia nuclei, these signals are output back to the cortex from the thalamus (Alexander et al. 1986; Haber 2016; Parent and Hazrati 1995). This suggests that the current results reflect a link between MRS and the output from thalamus to premotor and prefrontal regions that drive behavioural responses following integration and excitation or inhibition with the BG. However, this account is complicated by the existence of recurrent and lateral connections (e.g., from thalamus to striatum; Haber 2016). Therefore, future work should assess the link between MRS or music-induced pleasure and effective connectivity within CBGT circuits.

Finally, we did not collect measures of general reward sensitivity (e.g., the physical anhedonia scale). However, previous work suggests that general reward sensitivity and music reward sensitivity are distinct (Martínez-Molina et al. 2019; Mas-Herrero et al. 2014). Further, the range of MRS scores found here, including within the non-musician group, matched that in previous work (Martínez-Molina et al. 2019), while the significant fixels were only found in musicians who showed relatively high MRS overall. Therefore, general reward sensitivity, particularly that outside the normal range (e.g., due to general anhedonia), is unlikely to account for the current results.

## Conclusion

Here we expand on previous work investigating the neural correlates of MRS, extending the networks involved beyond auditory and reward regions to include motor and dorsal networks. This suggests a role of cognitive and motor processes in MRS and provides a potential structural basis for the functional activation within dorsal and motor networks seen during pleasurable music listening. The pleasure we derive from music is thought to rely on a complex array of processes including the generation and monitoring of sensory and reward-based predictions driven by the current musical context as well as long-term schemas and inference regarding the composer’s intentions (Salimpoor et al. 2015). This would necessitate the maintenance of dynamic representations of ongoing musical input as well as integration with longer-lasting representations, including autobiographical memories (Quinci et al. 2022). This work also adds to a growing body of literature highlighting the link between musical training and perceptual and affective processes, and the role of dorsal and motor networks in forming this link. In sum, this work supports the crucial role of motor and dorsal networks in musical pleasure, furthering our understanding of how music enriches our lives and with the potential to inform the use of music in clinical and therapeutic settings.

## Electronic supplementary material

Below is the link to the electronic supplementary material.

Supplementary Material 1

## Data Availability

The brain images generated during the current study are not publicly available due to privacy reasons but are available from the corresponding author on reasonable request.

## References

[CR1] Agosta S, Magnago D, Tyler S, Grossman E, Galante E, Ferraro F, Battelli L (2017) The pivotal role of the right parietal lobe in temporal attention. J Cogn Neurosci 29(5):805–815. 10.1162/jocn 27991181 10.1162/jocn_a_01086

[CR3] Albouy P, Weiss A, Baillet S, Zatorre RJ (2017) Selective entrainment of Theta oscillations in the dorsal Stream Causally enhances auditory Working Memory performance. Neuron 94(1):193–206e5. 10.1016/j.neuron.2017.03.015 28343866 10.1016/j.neuron.2017.03.015

[CR2] Albouy P, Benjamin L, Morillon B, Zatorre RJ (2020) Distinct sensitivity to spectrotemporal modulation supports brain asymmetry for speech and melody. Science 367(6481):16. 10.1126/science.1252826 32108113 10.1126/science.aaz3468

[CR4] Alexander G, DeLong MR, Strick PL (1986) Parallel Organization of functionally segregated circuits linking basal ganglia and cortex. Annu Rev Neurosci 9(1):357–381. 10.1146/annurev.neuro.9.1.357 3085570 10.1146/annurev.ne.09.030186.002041

[CR5] Alluri V, Toiviainen P, Burunat I, Kliuchko M, Vuust P, Brattico E (2017) Connectivity patterns during music listening: evidence for action-based processing in musicians. Hum Brain Mapp 38(6):2955–2970. 10.1002/hbm.23565 28349620 10.1002/hbm.23565 PMC6866725

[CR7] Andersson JLR, Sotiropoulos SN (2016) An integrated approach to correction for off-resonance effects and subject movement in diffusion MR imaging. NeuroImage 125:1063–1078. 10.1016/j.neuroimage.2015.10.019 26481672 10.1016/j.neuroimage.2015.10.019 PMC4692656

[CR6] Andersson JLR, Skare S, Ashburner J (2003) How to correct susceptibility distortions in spin-echo echo-planar images: application to diffusion tensor imaging. NeuroImage 20(2):870–888. 10.1016/S1053-8119(03)00336-7 14568458 10.1016/S1053-8119(03)00336-7

[CR8] Ara A, Marco-Pallarés J (2020) Fronto-temporal theta phase-synchronization underlies music-evoked pleasantness. NeuroImage 212(February):0–7. 10.1016/j.neuroimage.2020.116665 10.1016/j.neuroimage.2020.116665 32087373

[CR9] Ara A, Marco-Pallarés J (2021) Different theta connectivity patterns underlie pleasantness evoked by familiar and unfamiliar music. Sci Rep 11(1):1–9. 10.1038/s41598-021-98033-5 34535731 10.1038/s41598-021-98033-5 PMC8448873

[CR10] Araneda R, Renier L, Ebner-Karestinos D, Dricot L, De Volder AG (2016) Hearing, feeling or seeing a beat recruits a supramodal network in the auditory dorsal stream. Eur J Neurosci 45:1439–1450. 10.1017/CBO9781107415324.004 27471102 10.1111/ejn.13349

[CR11] Avants BB, Tustison NJ, Stauffer M, Song G, Wu B, Gee JC (2014) The insight ToolKit image registration framework. Front Neuroinformatics 8(APR):1–13. 10.3389/fninf.2014.00044 10.3389/fninf.2014.00044 PMC4009425 24817849

[CR12] Baer LH, Park MTM, Bailey Ja, Chakravarty MM, Li KZH, Penhune VB (2015) Regional cerebellar volumes are related to early musical training and finger tapping performance. NeuroImage 109:130–139. 10.1016/j.neuroimage.2014.12.076 25583606 10.1016/j.neuroimage.2014.12.076

[CR13] Bastiani M, Cottaar M, Fitzgibbon SP, Suri S, Alfaro-Almagro F, Sotiropoulos SN, Andersson JLR (2019) Automated quality control for within and between studies diffusion MRI data using a non-parametric framework for movement and distortion correction. NeuroImage 184May 2018:801–812. 10.1016/j.neuroimage.2018.09.073 10.1016/j.neuroimage.2018.09.073 PMC6264528 30267859

[CR14] Battelli L, Pascual-Leone A, Cavanagh P (2007) The when pathway of the right parietal lobe. Trends Cogn Sci 11(5):204–210. 10.1016/j.tics.2007.03.001 17379569 10.1016/j.tics.2007.03.001 PMC3613278

[CR15] Belfi AM, Evans E, Heskje J, Bruss J, Tranel D (2017) Musical anhedonia after focal brain damage. Neuropsychologia 97(January):29–37. 10.1016/j.neuropsychologia.2017.01.030 28159618 10.1016/j.neuropsychologia.2017.01.030

[CR16] Bengtsson SL, Nagy Z, Skare S, Forsman L, Forssberg H, Ullén F (2005) Extensive piano practicing has regionally specific effects on white matter development. Nat Neurosci 8(9):1148–1150. 10.1038/nn1516 16116456 10.1038/nn1516

[CR17] Bermudez P, Zatorre RJ (2005) Differences in gray matter between musicians and nonmusicians. Ann N Y Acad Sci 10602005:395–399. 10.1196/annals.1360.057 10.1196/annals.1360.057 16597791

[CR18] Bianco R, Novembre G, Keller PE, Seung-Goo K, Scharf F, Friederici A, Sammler D (2016) Neural networks for harmonic structure in music perception and action. NeuroImage 142:454–464. 10.1016/j.neuroimage.2016.08.025 27542722 10.1016/j.neuroimage.2016.08.025

[CR19] Bianco R, Novembre G, Ringer H, Kohler N, Keller PE, Villringer A, Sammler D (2021) Lateral prefrontal cortex is a hub for music production from structural rules to movements. Cereb Cortex 1–18. 10.1093/cercor/bhab454 10.1093/cercor/bhab454 PMC9476625 34965579

[CR20] Blecher T, Tal I, Ben-Shachar M (2016) White matter microstructural properties correlate with sensorimotor synchronization abilities. NeuroImage 138:1–12. 10.1016/j.neuroimage.2016.05.022 27165760 10.1016/j.neuroimage.2016.05.022

[CR21] Blood AJ, Zatorre RJ (2001) Intensely pleasurable responses to music correlate with activity in brain regions implicated in reward and emotion. Proc Natl Acad Sci USA 98(20):11818–11823. 10.1073/pnas.191355898 11573015 10.1073/pnas.191355898 PMC58814

[CR23] Brown S, Martinez MJ, Parsons LM (2004) Passive music listening spontaneously engages limbic and paralimbic systems. NeuroReport 15(13):2033–2037. 10.1097/00001756-200409150-00008 15486477 10.1097/00001756-200409150-00008

[CR22] Brown RM, Zatorre RJ, Penhune VB (2015) Expert music performance: cognitive, neural, and developmental bases. Prog Brain Res 57–86. 10.1016/bs.pbr.2014.11.021 10.1016/bs.pbr.2014.11.021 25725910

[CR24] Cannon JJ, Patel AD (2020) How beat perception co-opts Motor Neurophysiology. Trends Cogn Sci 25(2):137–150. 10.1016/j.tics.2020.11.002 33353800 10.1016/j.tics.2020.11.002 PMC9440376

[CR25] Céspedes-Guevara J, Dibben N (2022) The role of Embodied Simulation and Visual Imagery in Emotional Contagion with Music. Music Sci 5(122):1–27. 10.1177/20592043221093836

[CR26] Chabin T, Gabriel D, Chansophonkul T, Michelant L, Joucla C, Haffen E, Pazart L (2020) Cortical patterns of pleasurable musical chills revealed by high-density EEG. Front NeuroSci 14(November):1–11. 10.3389/fnins.2020.565815 33224021 10.3389/fnins.2020.565815 PMC7670092

[CR27] Chauvigné LaS, Gitau KM, Brown S (2014) The neural basis of audiomotor entrainment: an ALE meta-analysis. Front Hum Neurosci 8(September):776. 10.3389/fnhum.2014.00776 25324765 10.3389/fnhum.2014.00776 PMC4179708

[CR28] Chen JL, Penhune VB, Zatorre RJ (2008) Listening to musical rhythms recruits motor regions of the brain. Cereb Cortex 18(12):2844–2854. 10.1093/cercor/bhn042 18388350 10.1093/cercor/bhn042

[CR29] Cheng L, Lin Y, Yeh T, Tseng WI, Chen L (2022) Long-term musical training induces white matter plasticity in emotion and language networks. Hum Brain Mapp September 2020:1–13. 10.1002/hbm.26054 10.1002/hbm.26054 PMC9783470 36005832

[CR30] Cheung VKM, Harrison PMC, Meyer L, Pearce MT, Haynes J-D, Koelsch S (2019) Uncertainty and Surprise jointly predict musical pleasure and Amygdala, Hippocampus, and auditory cortex activity. Curr Biol 1–9. 10.1016/j.cub.2019.09.067 10.1016/j.cub.2019.09.067 31708393

[CR31] Coull JT, Davranche K, Nazarian B, Vidal F (2013) Functional anatomy of timing differs for production versus prediction of time intervals. Neuropsychologia 51(2):309–319. 10.1016/j.neuropsychologia.2012.08.017 22964490 10.1016/j.neuropsychologia.2012.08.017

[CR32] Criscuolo A, Pando-Naude V, Bonetti L, Vuust P, Brattico E (2022) An ALE meta-analytic review of musical expertise. Sci Rep 12(1):1–17. 10.1038/s41598-022-14959-4 35821035 10.1038/s41598-022-14959-4 PMC9276732

[CR33] de Manzano Ö, Ullén F (2018) Same genes, different brains: neuroanatomical differences between monozygotic twins discordant for musical training. Cereb Cortex 28(1):387–394. 10.1093/cercor/bhx299 29136105 10.1093/cercor/bhx299

[CR35] Dhollander T, Raffelt DA, Connelly A (2016) Unsupervised 3-tissue response function estimation from single-shell or multi-shell diffusion MR data without a co-registered T1 image Brain network disruption in chronic stroke patients View project Review of Fixel-Based Analysis (FBA) of diffusion MRI (. *ISMRM Workshop on Breaking the Barriers of Diffusion MRI*, (September), 1–2. Retrieved from https://www.researchgate.net/publication/307863133

[CR34] Dhollander T, Clemente A, Singh M, Boonstra F, Civier O, Duque JD, Caeyenberghs K (2021) Fixel-based analysis of Diffusion MRI: methods, applications, challenges and opportunities. NeuroImage 241(July):118417. 10.1016/j.neuroimage.2021.118417 34298083 10.1016/j.neuroimage.2021.118417

[CR36] Di Liberto GM, Pelofi C, Bianco R, Patel P, Mehta AD, Herrero JL, Mesgarani N (2020) Cortical encoding of melodic expectations in human temporal cortex. ELife 9:1–26. 10.7554/eLife.51784 10.7554/eLife.51784 PMC7053998 32122465

[CR37] Farquharson S, Tournier JD, Calamante F, Fabinyi G, Schneider-Kolsky M, Jackson GD, Connelly A (2013) White matter fiber tractography: why we need to move beyond DTI. J Neurosurg 118(6):1367–1377. 10.3171/2013.2.JNS121294 23540269 10.3171/2013.2.JNS121294

[CR38] Foster NEV, Halpern AR, Zatorre RJ (2013) Common parietal activation in musical mental transformations across pitch and time. NeuroImage 75:27–35 23470983 10.1016/j.neuroimage.2013.02.044

[CR39] Freitas C, Manzato E, Burini A, Taylor MJ, Lerch JP, Anagnostou E (2018) Neural correlates of familiarity in music listening: a systematic review and a neuroimaging meta-analysis. Front NeuroSci 12(OCT):1–14. 10.3389/fnins.2018.00686 30344470 10.3389/fnins.2018.00686 PMC6183416

[CR40] Fuelscher I, Hyde C, Anderson V, Silk TJ (2021) White matter tract signatures of fiber density and morphology in ADHD. Cortex 138:329–340. 10.1016/j.cortex.2021.02.015 33784515 10.1016/j.cortex.2021.02.015

[CR41] Gatti D, Rinaldi L, Ferreri L, Vecchi T (2021) The human cerebellum as a hub of the predictive brain. Brain Sci 11(11):1–10. 10.3390/brainsci11111492 10.3390/brainsci11111492 PMC8615481 34827491

[CR42] Gelding RW, Thompson WF, Johnson BW (2019) Musical imagery depends upon coordination of auditory and sensorimotor brain activity. Sci Rep 9(1):16823. 10.1038/s41598-019-53260-9 31727968 10.1038/s41598-019-53260-9 PMC6856354

[CR43] Giacosa C, Karpati FJ, Foster NEV, Penhune VB, Hyde KL (2016) Dance and music training have different effects on white matter diffusivity in sensorimotor pathways. NeuroImage 135:273–286. 10.1016/j.neuroimage.2016.04.048 27114054 10.1016/j.neuroimage.2016.04.048

[CR44] Gold BP, Mas-herrero E, Zeighami Y, Benovoy M, Dagher A, Zatorre RJ (2019) Musical reward prediction errors engage the nucleus accumbens and motivate learning. *PNAS*, 1–6. 10.1073/pnas.1809855116 10.1073/pnas.1809855116 PMC6386687 30728301

[CR45] Gold BP, Pearce MT, Mcintosh AR, Chang C, Dagher A, Zatorre RJ (2023) Auditory and reward structures reflect the pleasure of musical expectancies during naturalistic listening. Front NeuroSci 17(1209398). 10.3389/fnins.2023.1209398 10.3389/fnins.2023.1209398 PMC10625409 37928727

[CR46] Grahn JA, Brett M (2007) Rhythm and beat perception in motor areas of the brain. J Cogn Neurosci 19(5):893–906. 10.1162/jocn.2007.19.5.893 17488212 10.1162/jocn.2007.19.5.893

[CR47] Grahn JA, Rowe JB (2009) Feeling the beat: premotor and striatal interactions in musicians and nonmusicians during beat perception. J Neurosci 29(23):7540–7548. 10.1523/JNEUROSCI.2018-08.2009 19515922 10.1523/JNEUROSCI.2018-08.2009 PMC2702750

[CR48] Green AC, Bærentsen KB, Stodkilde-Jorgensen H, Roepstorff A, Vuust P (2012) Listen, learn, like! Dorsolateral prefrontal cortex involved in the mere exposure effect in music. Neurol Res Int 2012. 10.1155/2012/846270 10.1155/2012/846270 PMC3324153 22548168

[CR49] Groussard M, La Joie R, Rauchs G, Landeau B, Chételat G, Viader F, Platel H (2010) When music and long-term memory interact: effects of musical expertise on functional and structural plasticity in the hippocampus. PLoS ONE 5(10):1–8. 10.1371/journal.pone.0013225 10.1371/journal.pone.0013225 PMC2950159 20957158

[CR50] Haber SN (2016) Corticostriatal circuitry. Dialog Clin Neurosci 18:7–21 10.31887/DCNS.2016.18.1/shaber PMC4826773 27069376

[CR51] Halwani GF, Loui P, Rüber T, Schlaug G (2011) Effects of practice and experience on the arcuate fasciculus: comparing singers, instrumentalists, and non-musicians. Front Psychol 2(July):156. 10.3389/fpsyg.2011.00156 21779271 10.3389/fpsyg.2011.00156 PMC3133864

[CR52] Han Y, Yang H, Lv YT, Zhu CZ, He Y, Tang HH, Dong Q (2009) Gray Matter density and white matter integrity in pianists’ brain: a combined structural and diffusion tensor MRI study. Neurosci Lett 459(1):3–6. 10.1016/j.neulet.2008.07.056 18672026 10.1016/j.neulet.2008.07.056

[CR53] Hansen NC, Højlund A, Møller C, Pearce M, Vuust P (2022) Musicians show more integrated neural processing of contextually relevant acoustic features. Front NeuroSci 16(October):1–18. 10.3389/fnins.2022.907540 10.3389/fnins.2022.907540 PMC9612920 36312026

[CR54] Harvey PO, Pruessner J, Czechowska Y, Lepage M (2007) Individual differences in trait anhedonia: a structural and functional magnetic resonance imaging study in non-clinical subjects. Mol Psychiatry 12(8):767–775. 10.1038/sj.mp.4002021 10.1038/sj.mp.4002021 17505465

[CR55] Herholz SC, Halpern AR, Zatorre RJ (2012) Neuronal correlates of perception, imagery, and memory for familiar tunes. J Cogn Neurosci 24(6):1382–1397. 10.1162/jocn_a_00216 22360595 10.1162/jocn_a_00216

[CR56] Hernández M, Palomar-García MÁ, Nohales-Nieto B, Olcina-Sempere G, Villar-Rodríguez E, Pastor R, Parcet MA (2019) Separate contribution of striatum volume and pitch discrimination to individual differences in music reward. Psychol Sci 30(9):1352–1361. 10.1177/0956797619859339 31340130 10.1177/0956797619859339

[CR57] Hickok G, Poeppel D (2007) The Cortical Organization of Speech Processing. Nat Rev Neurosci 8:393–402. 10.1038/nrn2113 17431404 10.1038/nrn2113

[CR58] Hickok G, Poeppel D (2016) Neural Basis of Speech Perception. In *Neurobiology of Language* (pp. 299–310). Elsevier Inc. 10.1016/B978-0-12-407794-2.00025-0

[CR59] Holland D, Kuperman JM, Dale AM (2010) Efficient correction of inhomogeneous static magnetic field-induced distortion in Echo Planar Imaging. NeuroImage 50(1):175–183. 10.1016/j.neuroimage.2009.11.044 19944768 10.1016/j.neuroimage.2009.11.044 PMC2819607

[CR60] Huron D (2006) *Sweet Anticipation: Music and the Psychology of Expectation*. *MIT Press*. Cambridge, MA. 10.1525/mp.2007.24.5.511

[CR61] Imfeld A, Oechslin MS, Meyer M, Loenneker T, Jancke L (2009) White matter plasticity in the corticospinal tract of musicians: a diffusion tensor imaging study. NeuroImage 46(3):600–607. 10.1016/j.neuroimage.2009.02.025 19264144 10.1016/j.neuroimage.2009.02.025

[CR62] Jagiello R, Pomper U, Yoneya M, Zhao S, Chait M (2019) Rapid brain responses to familiar vs. unfamiliar music - an EEG and pupillometry study. Sci Rep 9:466359. 10.1101/466359 10.1038/s41598-019-51759-9 PMC6821741 31666553

[CR63] Jenkinson M, Beckmann CF, Behrens TEJ, Woolrich MW, Smith SM (2012) FSL NeuroImage 62:782–790. 10.1016/j.neuroimage.2011.09.015 21979382 10.1016/j.neuroimage.2011.09.015

[CR64] Jeurissen B, Leemans A, Tournier JD, Jones DK, Sijbers J (2013) Investigating the prevalence of complex fiber configurations in white matter tissue with diffusion magnetic resonance imaging. Hum Brain Mapp 34(11):2747–2766. 10.1002/hbm.22099 22611035 10.1002/hbm.22099 PMC6870534

[CR65] Jiang L, Zhang R, Tao L, Zhang Y, Zhou Y, Cai Q (2023) Neural mechanisms of musical syntax and tonality, and the effect of musicianship. Front Psychol 14:1092051. 10.3389/fpsyg.2023.1092051 36844277 10.3389/fpsyg.2023.1092051 PMC9948014

[CR66] Johnsen EL, Tranel D, Lutgendorf S, Adolphs R (2009) A neuroanatomical dissociation for emotion induced by music. Int J Psychophysiol 72(1):24–33. 10.1016/j.ijpsycho.2008.03.011 18824047 10.1016/j.ijpsycho.2008.03.011 PMC2656600

[CR67] Jones DK (2010) Diffusion mri. Oxford University Press

[CR68] Jones DK, Knösche TR, Turner R (2013) White matter integrity, fiber count, and other fallacies: the do’s and don’ts of diffusion MRI. NeuroImage 73:239–254. 10.1016/j.neuroimage.2012.06.081 22846632 10.1016/j.neuroimage.2012.06.081

[CR69] Jongsma MLA, Desain P, Honing H (2004) Rhythmic context influences the auditory evoked potentials of musicians and nonmusicians. Biol Psychol 66(2):129–152. 10.1016/j.biopsycho.2003.10.002 15041136 10.1016/j.biopsycho.2003.10.002

[CR70] Jongsma MLA, Eichele T, Quiroga RQ, Jenks KM, Desain P, Honing H, Van Rijn CM (2005) Expectancy effects on omission evoked potentials in musicians and non-musicians. Psychophysiology 42(2):191–201. 10.1111/j.1469-8986.2005.00269.x 15787856 10.1111/j.1469-8986.2005.00269.x

[CR71] Juslin PN (2013) From everyday emotions to aesthetic emotions: towards a unified theory of musical emotions. Phys Life Rev 10(3):235–266. 10.1016/j.plrev.2013.05.008 23769678 10.1016/j.plrev.2013.05.008

[CR72] Karabanov A, Blom O, Forsman L, Ullén F (2009) The dorsal auditory pathway is involved in performance of both visual and auditory rhythms. NeuroImage 44(2):480–488. 10.1016/j.neuroimage.2008.08.047 18848999 10.1016/j.neuroimage.2008.08.047

[CR73] Kasdan AV, Burgess AN, Pizzagalli F, Scartozzi A, Chern A, Kotz SA, Gordon RL (2022) Identifying a brain network for musical rhythm: a functional neuroimaging meta-analysis and systematic review. Neurosci Biobehavioral Reviews 136:104588. 10.1016/j.neubiorev.2022.104588 10.1016/j.neubiorev.2022.104588 PMC9195154 35259422

[CR74] Kellner E, Dhital B, Kiselev VG, Reisert M (2016) Gibbs-ringing artifact removal based on local subvoxel-shifts. Magn Reson Med 76(5):1574–1581. 10.1002/mrm.26054 26745823 10.1002/mrm.26054

[CR76] Koelsch S, Vuust P, Friston K (2019) Predictive processes and the Peculiar Case of Music. Trends Cogn Sci 23(1):63–77. 10.1016/j.tics.2018.10.006 30471869 10.1016/j.tics.2018.10.006

[CR75] Koelsch S, Cheung VKM, Jentschke S, Haynes JD (2021) Neocortical substrates of feelings evoked with music in the ACC, insula, and somatosensory cortex. Sci Rep 11(1):1–11. 10.1038/s41598-021-89405-y 33980876 10.1038/s41598-021-89405-y PMC8115666

[CR77] Konoike N, Kotozaki Y, Jeong H, Miyazaki A, Sakaki K, Shinada T, Nakamura K (2015) Temporal and motor representation of rhythm in fronto-parietal cortical areas: an fMRI study. PLoS ONE 10(6):1–19. 10.1371/journal.pone.0130120 10.1371/journal.pone.0130120 PMC4468110 26076024

[CR78] Kornysheva K, von Cramon DY, Jacobsen T, Schubotz RI (2010) Tuning-in to the beat: aesthetic appreciation of musical rhythms correlates with a Premotor Activity Boost. Hum Brain Mapp 31:48–64. 10.1002/hbm.20844 19585590 10.1002/hbm.20844 PMC6870655

[CR79] Kumar S, Joseph S, Gander PE, Barascud N, Halpern AR, Griffiths TD (2016) A brain system for auditory working memory. J Neurosci 36(16):4492–4505. 10.1523/JNEUROSCI.4341-14.2016 27098693 10.1523/JNEUROSCI.4341-14.2016 PMC4837683

[CR80] Kung S-J, Chen JL, Zatorre RJ, Penhune VB (2013) Interacting cortical and basal ganglia networks underlying finding and tapping to the musical beat. J Cogn Neurosci 25(3):401–420. 10.1162/jocn_a_00325 23163420 10.1162/jocn_a_00325

[CR82] Lappe C, Steinsträter O, Pantev C (2013) Rhythmic and melodic deviations in musical sequences recruit different cortical areas for mismatch detection. Front Hum Neurosci 7(June):260. 10.3389/fnhum.2013.00260 23759929 10.3389/fnhum.2013.00260 PMC3675320

[CR81] Lappe C, Lappe M, Pantev C (2016) Differential processing of melodic, rhythmic and simple tone deviations in musicians -an MEG study. NeuroImage 124:898–905. 10.1016/j.neuroimage.2015.09.059 26436712 10.1016/j.neuroimage.2015.09.059

[CR83] Leipold S, Klein C, Jäncke L (2021) Musical expertise shapes functional and structural brain networks independent of absolute pitch ability. J Neurosci November 2020(JN–RM–1985–20). 10.1523/jneurosci.1985-20.2020 10.1523/JNEUROSCI.1985-20.2020 PMC7984587 33495199

[CR84] Leman M, Maes P, Nijs L, Van Dyck E (2018) What is embodied music cognition. In B. R. (Ed.), Springer Handbook of systematic musicology. Springer Handbooks. Springer, Berlin, Heidelberg, Berlin, Heidelberg, pp 747–760

[CR85] Li CW, Guo FY, Tsai CG (2021) Predictive processing, cognitive control, and tonality stability of music: an fMRI study of chromatic harmony. Brain Cogn 151(1):105751. 10.1016/j.bandc.2021.105751 33991840 10.1016/j.bandc.2021.105751

[CR86] Loui P, Alsop D, Schlaug G (2009) Tone deafness: a new disconnection syndrome? J Neurosci 29(33):10215–10220. 10.1523/JNEUROSCI.1701-09.2009 19692596 10.1523/JNEUROSCI.1701-09.2009 PMC2747525

[CR87] Loui P, Patterson S, Sachs ME, Leung Y, Zeng T, Przysinda E (2017) White matter correlates of musical Anhedonia: implications for evolution of music. Front Psychol 8(SEP):1–10. 10.3389/fpsyg.2017.01664 28993748 10.3389/fpsyg.2017.01664 PMC5622186

[CR88] Lumaca M, Baggio G, Vuust P (2021) White matter variability in auditory callosal pathways contributes to variation in the cultural transmission of auditory symbolic systems. Brain Struct Function 226(6):1943–1959. 10.1007/s00429-021-02302-y 10.1007/s00429-021-02302-y 34050791

[CR89] Luo C, Guo Zwei, Lai Y, xiu, Liao W, Liu Q, Kendrick KM, Li H (2012) Musical training induces functional plasticity in perceptual and motor networks: insights from resting-state fMRI. PLoS ONE 7(5):1–10. 10.1371/journal.pone.0036568 10.1371/journal.pone.0036568 PMC3346725 22586478

[CR90] Martínez-Molina N, Mas-Herrero E, Rodríguez-Fornells A, Zatorre RJ, Marco-Pallarés J (2016) Neural correlates of specific musical anhedonia. PNAS, E7337–E7345. 10.1073/PNAS.1611211113 10.1073/pnas.1611211113 PMC5135354 27799544

[CR91] Martínez-Molina N, Mas-Herrero E, Rodríguez-Fornells A, Zatorre RJ, Marco-Pallarés J (2019) White matter microstructure reflects individual differences in music reward sensitivity. J Neurosci 39(25):5018–5027 31000588 10.1523/JNEUROSCI.2020-18.2019 PMC6670256

[CR94] Mas-Herrero E, Marco-Pallarés J, Lorenzo-Seva U, Zatorre RJ, Rodriguez-Fornells A (2013) Individual differences in music reward experiences. Music Percept 31(2):118–138

[CR95] Mas-Herrero E, Zatorre RJ, Rodriguez-Fornells A, Marco-Pallarés J (2014) Dissociation between musical and monetary reward responses in specific musical anhedonia. Curr Biol 24(6):699–704. 10.1016/j.cub.2014.01.068 24613311 10.1016/j.cub.2014.01.068

[CR92] Mas-Herrero E, Dagher A, Zatorre RJ (2018) Modulating musical reward sensitivity up and down with transcranial magnetic stimulation. Nat Hum Behav 2:27–32. 10.1038/s41562-017-0241-z 30980048 10.1038/s41562-017-0241-z

[CR93] Mas-Herrero E, Maini L, Sescousse G, Zatorre RJ (2021) Common and distinct neural correlates of music and food-induced pleasure: a coordinate-based meta-analysis of neuroimaging studies. Neurosci Biobehav Rev 123(January):61–71. 10.1016/j.neubiorev.2020.12.008 33440196 10.1016/j.neubiorev.2020.12.008

[CR96] Masina F, Pezzetta R, Lago S, Mantini D, Scarpazza C, Arcara G (2022) Disconnection from prediction: a systematic review on the role of right temporoparietal junction in aberrant predictive processing. Neurosci Biobehav Rev 138(January):104713. 10.1016/j.neubiorev.2022.104713 35636560 10.1016/j.neubiorev.2022.104713

[CR97] Matthews TE, Witek MAG, Heggli OA, Penhune VB, Vuust P (2019) The sensation of groove is affected by the interaction of rhythmic and harmonic complexity. PLoS ONE 14(1):1–17. 10.1371/journal.pone.0204539 10.1371/journal.pone.0204539 PMC6328141 30629596

[CR98] Matthews TE, Witek MAG, Lund T, Vuust P, Penhune VB (2020) The sensation of groove engages motor and reward networks. NeuroImage 214(116768):1–12. 10.1016/j.neuroimage.2020.116768 10.1016/j.neuroimage.2020.116768 32217163

[CR99] Matthews TE, Witek MAG, Thibodeau JLN, Vuust P, Penhune VB (2022) Perceived motor synchrony with the beat is more strongly related to groove than measured synchrony. Music Percept 39(5):423–442

[CR100] Mazzoni M, Moretti P, Pardossi L, Vista M, Muratorio A (1993) Letters to the editor. J Neurol Neurosurg Psychiatry 56:322–324 8459254 10.1136/jnnp.56.3.322 PMC1014874

[CR101] Menon V, Levitin DJ (2005) The rewards of music listening: response and physiological connectivity of the mesolimbic system. NeuroImage 28:175–184. 10.1016/j.neuroimage.2005.05.053 16023376 10.1016/j.neuroimage.2005.05.053

[CR102] Merchant H, Honing H (2014) Are non-human primates capable of rhythmic entrainment? Evidence for the gradual audiomotor evolution hypothesis. Front NeuroSci 7(January):1–8. 10.3389/fnins.2014.00274 10.3389/fnins.2013.00274 PMC3894452 24478618

[CR103] Meyer LB (1956) Emotion and meaning in music. University of Chicago Press, Chicago

[CR104] Mosing MA, Madison G, Pedersen NL, Ullén F (2016) Investigating cognitive transfer within the framework of music practice: genetic pleiotropy rather than causality. Dev Sci 19(3):504–512. 10.1111/desc.12306 25939545 10.1111/desc.12306

[CR106] Oechslin MS, Imfeld A, Loenneker T, Meyer M, Jäncke L (2010) The plasticity of the superior longitudinal fasciculus as a function of musical expertise: a diffusion tensor imaging study. Front Hum Neurosci 3(FEB):1–12. 10.3389/neuro.09.076.2009 10.3389/neuro.09.076.2009 PMC2821183 20161812

[CR105] Oechslin MS, Gschwind M, James CE (2018) Tracking training-related plasticity by combining fMRI and DTI: the right hemisphere ventral Stream mediates Musical Syntax Processing. Cereb Cortex 28(4):1209–1218. 10.1093/cercor/bhx033 28203797 10.1093/cercor/bhx033

[CR107] Oestreich LKL, Randeniya R, Garrido MI (2019) Auditory white matter pathways are associated with effective connectivity of auditory prediction errors within a fronto-temporal network. NeuroImage 195(April):454–462. 10.1016/j.neuroimage.2019.04.008 30959193 10.1016/j.neuroimage.2019.04.008

[CR108] Palmer C, Krumhansl CL (1990) Mental representations for Musical Meter. J Exp Psychol Hum Percept Perform 16(4):728–741. 10.1037/0096-1523.16.4.728 2148588 10.1037//0096-1523.16.4.728

[CR109] Pando-Naude V, Patyczek A, Bonetti L, Vuust P (2021) An ALE meta–analytic review of top–down and bottom–up processing of music in the brain. Sci Rep 11(1):20813. 10.1038/s41598-021-00139-3 34675231 10.1038/s41598-021-00139-3 PMC8531391

[CR110] Parent A, Hazrati L-N (1995) Functional anatomy of the basal ganglia. I. The cortico-basal ganglia-thalamo-cortical loop. Brain Res Rev 20:91–127. 10.1097/01376517-200010000-00002 7711769 10.1016/0165-0173(94)00007-c

[CR111] Pearce MT, Wiggins GA (2012) Auditory expectation: the information dynamics of music perception and cognition. Top Cogn Sci 4(4):625–652. 10.1111/j.1756-8765.2012.01214.x 22847872 10.1111/j.1756-8765.2012.01214.x

[CR112] Pesnot Lerousseau J, Schön D (2021) Musical expertise is Associated with improved neural statistical learning in the Auditory Domain. Cereb Cortex 1–14. 10.1093/cercor/bhab128 10.1093/cercor/bhab128 34013316

[CR113] Quinci MA, Belden A, Goutama V, Gong D, Hanser S, Donovan NJ, Loui P (2022) Longitudinal changes in auditory and reward systems following receptive music-based intervention in older adults. Sci Rep 12(1):1–15. 10.1038/s41598-022-15687-5 35798784 10.1038/s41598-022-15687-5 PMC9261172

[CR114] Quiroga-Martinez DR, Hansen NC, Højlund A, Pearce M, Brattico E, Vuust P (2020) Musical prediction error responses similarly reduced by predictive uncertainty in musicians and non-musicians. Eur J Neurosci 51:2250–2269 31891423 10.1111/ejn.14667

[CR117] Raffelt DA, Tournier JD, Fripp J, Crozier S, Connelly A, Salvado O (2011) Symmetric diffeomorphic registration of fibre orientation distributions. NeuroImage 56(3):1171–1180. 10.1016/j.neuroimage.2011.02.014 21316463 10.1016/j.neuroimage.2011.02.014

[CR115] Raffelt DA, Smith RE, Ridgway GR, Tournier JD, Vaughan DN, Rose S, Connelly A (2015) Connectivity-based fixel enhancement: whole-brain statistical analysis of diffusion MRI measures in the presence of crossing fibres. NeuroImage 117:40–55. 10.1016/j.neuroimage.2015.05.039 26004503 10.1016/j.neuroimage.2015.05.039 PMC4528070

[CR116] Raffelt DA, Tournier J-D, Smith RE, Dhollander T, Tournier J-D, Tabbara R, Connelly A (2017a) Bias Field Correction and Intensity Normalisation for Quantitative Analysis of Apparent Fibre Density. *Proceedings of the International Society for Magnetic Resonance in Medicine*, (April), 3541. Retrieved from https://www.researchgate.net/publication/315836355

[CR118] Raffelt DA, Tournier JD, Smith RE, Vaughan DN, Jackson G, Ridgway GR, Connelly A (2017b) Investigating white matter fibre density and morphology using fixel-based analysis. NeuroImage 144:58–73. 10.1016/j.neuroimage.2016.09.029 27639350 10.1016/j.neuroimage.2016.09.029 PMC5182031

[CR119] Rauschecker JP, Scott SK (2009) Maps and streams in the auditory cortex: nonhuman primates illuminate human speech processing. Nat Neurosci 12(6):718–725. 10.1038/nn.2331 19471271 10.1038/nn.2331 PMC2846110

[CR120] Rigoulot S, Pell MD, Armony JL (2015) Time course of the influence of musical expertise on the processing of vocal and musical sounds. Neuroscience 290:175–184. 10.1016/j.neuroscience.2015.01.033 25637804 10.1016/j.neuroscience.2015.01.033

[CR121] Rizzolatti G, Craighero L (2004) The mirror-neuron system. Annu Rev Neurosci 27:169–192. 10.1146/annurev.neuro.27.070203.144230 15217330 10.1146/annurev.neuro.27.070203.144230

[CR122] Rogenmoser L, Zollinger N, Elmer S, Jäncke L (2016) Independent component processes underlying emotions during natural music listening. Soc Cognit Affect Neurosci 11(9):1428–1439. 10.1093/scan/nsw048 27217116 10.1093/scan/nsw048 PMC5015797

[CR123] Sachs ME, Ellis RJ, Schlaug G, Loui P (2016) Brain connectivity reflects human aesthetic responses to music. Soc Cognit Affect Neurosci 11(6):884–891. 10.1093/scan/nsw009 26966157 10.1093/scan/nsw009 PMC4884308

[CR124] Salimpoor VN, Benovoy M, Larcher K, Dagher A, Zatorre RJ (2011) Anatomically distinct dopamine release during anticipation and experience of peak emotion to music. Nat Neurosci 14(2):257–262. 10.1038/nn.2726 21217764 10.1038/nn.2726

[CR125] Salimpoor VN, van den Bosch I, Kovacevic N, McIntosh AR, Dagher A, Zatorre RJ (2013) Interactions between the nucleus accumbens and auditory cortices predict music reward value. Sci (New York N Y) 340(6129):216–219. 10.1126/science.1231059 10.1126/science.1231059 23580531

[CR126] Salimpoor VN, Zald DH, Zatorre RJ, Dagher A, McIntosh AR (2015) Predictions and the brain: how musical sounds become rewarding. Trends Cogn Sci 19(2):86–91. 10.1016/j.tics.2014.12.001 25534332 10.1016/j.tics.2014.12.001

[CR127] Satoh M, Nakase T, Nagata K, Tomimoto H (2011) Musical anhedonia: selective loss of emotional experience in listening to music. Neurocase 17(5):410–417. 10.1080/13554794.2010.532139 21714738 10.1080/13554794.2010.532139

[CR128] Scarr S, Mccartney K (1983) How people make their own environments: a theory of genotype → Environment effects. Child Dev 54(2):424–435 6683622 10.1111/j.1467-8624.1983.tb03884.x

[CR129] Schiffer A-M, Schubotz RI (2011) Caudate nucleus signals for breaches of expectation in a movement observation paradigm. Front Hum Neurosci 5(April):38. 10.3389/fnhum.2011.00038 21519392 10.3389/fnhum.2011.00038 PMC3078751

[CR130] Schubotz RI (2007) Prediction of external events with our motor system: towards a new framework. Trends Cogn Sci 11(5):211–218. 10.1016/j.tics.2007.02.006 17383218 10.1016/j.tics.2007.02.006

[CR131] Schubotz RI, Friederici A, von Cramon DY (2000) Time perception and motor timing: a common cortical and subcortical basis revealed by fMRI. NeuroImage 11(1):1–12. 10.1006/nimg.1999.0514 10686112 10.1006/nimg.1999.0514

[CR132] Schwartze M, Kotz SA (2013) A dual-pathway neural architecture for specific temporal prediction. Neurosci Biobehav Rev 37(10 Pt 2):2587–2596. 10.1016/j.neubiorev.2013.08.005 23994272 10.1016/j.neubiorev.2013.08.005

[CR133] Seger CA, Spiering BJ, Sares AG, Quraini SI, Alpeter C, David J, Thaut MH (2013) Corticostriatal contributions to musical expectancy perception. J Cogn Neurosci 25(7):1062–1077. 10.1162/jocn_a_00371 23410032 10.1162/jocn_a_00371

[CR134] Shany O, Singer N, Gold BP, Jacoby N, Tarrasch R, Hendler T, Granot R (2019) Surprise-related activation in the nucleus accumbens interacts with music-induced pleasantness. Soc Cognit Affect Neurosci 1–12. 10.1093/scan/nsz019 10.1093/scan/nsz019 PMC6523415 30892654

[CR135] Siman-Tov T, Granot RY, Shany O, Singer N, Gordon CR (2019) Is there a prediction network? Meta-analytic evidence for a cortical-subcortical network likely subserving prediction. Neurosci Biobehav Rev 105:262–275. 10.1016/j.neubiorev.2019.08.012 31437478 10.1016/j.neubiorev.2019.08.012

[CR136] Singer N, Jacobi N, Lin T, Raz G, Shpigelman L, Gilam G, Hendler T (2016) Common modulation of limbic network activation underlies the unfolding of musical emotions and its temporal attributes. NeuroImage 141:517–529. 10.1016/j.neuroimage.2016.07.002 27389788 10.1016/j.neuroimage.2016.07.002

[CR137] Smith RE, Tournier JD, Calamante F, Connelly A (2013) SIFT: spherical-deconvolution informed filtering of tractograms. NeuroImage 67:298–312. 10.1016/j.neuroimage.2012.11.049 23238430 10.1016/j.neuroimage.2012.11.049

[CR139] Steele CJ, Scholz J, Douaud G, Johansen-Berg H, Penhune VB (2012) Structural correlates of skilled performance on a motor sequence task. *Frontiers in Human Neuroscience*, *6*(OCTOBER 2012), 1–9. 10.3389/fnhum.2012.00289 10.3389/fnhum.2012.00289 PMC3486688 23125826

[CR138] Steele CJ, Bailey JA, Zatorre RJ, Penhune VB (2013) Early musical training and white-matter plasticity in the corpus callosum: evidence for a sensitive period. J Neurosci 33(3):1282–1290. 10.1523/JNEUROSCI.3578-12.2013 23325263 10.1523/JNEUROSCI.3578-12.2013 PMC6704889

[CR140] Stupacher J, Matthews TE, Pando-naude V, Foster O, Elst V, Vuust P (2022) The sweet spot between predictability and surprise: musical groove in brain, body, and social interactions. Front Psychol August1–9. 10.3389/fpsyg.2022.906190 10.3389/fpsyg.2022.906190 PMC9396343 36017431

[CR141] Tan YT, McPherson GE, Peretz I, Berkovic SF, Wilson SJ (2014) The genetic basis of music ability. Front Psychol 5(JUN):1–19. 10.3389/fpsyg.2014.00658 25018744 10.3389/fpsyg.2014.00658 PMC4073543

[CR142] Teki S, Griffiths TD (2016) Neural basis of Working Memory for Time intervals. Front NeuroSci 10(239):1–13. 10.1016/j.sbspro.2014.02.405 27313506 10.3389/fnins.2016.00239 PMC4888525

[CR143] Thaut M, Trimarchi P, Parsons L (2014) Human brain basis of Musical Rhythm Perception: common and distinct neural substrates for Meter, Tempo, and Pattern. Brain Sci 4(2):428–452. 10.3390/brainsci4020428 24961770 10.3390/brainsci4020428 PMC4101486

[CR144] Toiviainen P, Burunat I, Brattico E, Vuust P, Alluri V (2019) The chronnectome of musical beat. NeuroImage 216:1–13. 10.1016/j.neuroimage.2019.116191 10.1016/j.neuroimage.2019.116191 31525500

[CR145] Tournier JD, Calamante F, Connelly A (2007) Robust determination of the fibre orientation distribution in diffusion MRI: non-negativity constrained super-resolved spherical deconvolution. NeuroImage 35(4):1459–1472. 10.1016/j.neuroimage.2007.02.016 17379540 10.1016/j.neuroimage.2007.02.016

[CR146] Tournier JD, Smith R, Raffelt D, Tabbara R, Dhollander T, Pietsch M, Connelly A (2019) MRtrix3: a fast, flexible and open software framework for medical image processing and visualisation. NeuroImage 202(August):116137. 10.1016/j.neuroimage.2019.116137 31473352 10.1016/j.neuroimage.2019.116137

[CR147] Tustison NJ, Avants BB, Cook PA, Zheng Y, Egan A, Yushkevich PA, Gee JC (2010) N4ITK: improved N3 bias correction. IEEE Trans Med Imaging 29(6):1310–1320. 10.1109/TMI.2010.2046908 20378467 10.1109/TMI.2010.2046908 PMC3071855

[CR148] Vaquero L, Hartmann K, Ripollés P, Rojo N, Sierpowska J, François C, Altenmüller E (2015) Structural neuroplasticity in expert pianists depends on the age of musical training onset. NeuroImage. 10.1016/j.neuroimage.2015.11.008 26584868 10.1016/j.neuroimage.2015.11.008

[CR149] Vaquero L, Ramos-Escobar N, Cucurell D, François C, Putkinen V, Segura E, Rodríguez-Fornells A (2021) Arcuate Fasciculus Architecture is Associated with Individual differences in pre-attentive detection of unpredicted music changes. NeuroImage 229(December 2020):117759. 10.1016/j.neuroimage.2021.117759 33454403 10.1016/j.neuroimage.2021.117759

[CR150] Veraart J, Novikov DS, Christiaens D, Ades-aron B, Sijbers J, Fieremans E (2016) Denoising of diffusion MRI using random matrix theory. NeuroImage 142:394–406. 10.1016/j.neuroimage.2016.08.016 27523449 10.1016/j.neuroimage.2016.08.016 PMC5159209

[CR151] Vollmann H, Ragert P, Conde V, Villringer A, Classen J, Witte OW, Steele CJ (2014) Instrument specific use-dependent plasticity shapes the anatomical properties of the corpus callosum: a comparison between musicians and non-musicians. Front Behav Neurosci 8(JULY):1–8. 10.3389/fnbeh.2014.00245 25076879 10.3389/fnbeh.2014.00245 PMC4100438

[CR152] Vuong V, Hewan P, Perron M, Thaut MH, Alain C (2023) The neural bases of familiar music listening in healthy individuals: an activation likelihood estimation meta-analysis. Neurosci Biobehavioral Reviews 127120. 10.1016/j.neubiorev.2023.105423 10.1016/j.neubiorev.2023.105423 37839672

[CR157] Vuust P, Witek MAG (2014) Rhythmic complexity and predictive coding: a novel approach to modeling rhythm and meter perception in music. Front Psychol 5(1111):1–14. 10.3389/fpsyg.2014.01111 25324813 10.3389/fpsyg.2014.01111 PMC4181238

[CR156] Vuust P, Pallesen KJ, Bailey C, van Zuijen TL, Gjedde A, Roepstorff A, Østergaard L (2005) To musicians, the message is in the meter: pre-attentive neuronal responses to incongruent rhythm are left-lateralized in musicians. NeuroImage 24(2):560–564. 10.1016/j.neuroimage.2004.08.039 15627598 10.1016/j.neuroimage.2004.08.039

[CR155] Vuust P, Ostergaard L, Pallesen KJ, Bailey C, Roepstorff A (2009) Predictive coding of music -brain responses to rhythmic incongruity. Cortex 45(1):80–92. 10.1016/j.cortex.2008.05.014 19054506 10.1016/j.cortex.2008.05.014

[CR153] Vuust P, Brattico E, Seppänen M, Näätänen R, Tervaniemi M (2012) The sound of music: differentiating musicians using a fast, musical multi-feature mismatch negativity paradigm. Neuropsychologia 50(7):1432–1443. 10.1016/j.neuropsychologia.2012.02.028 22414595 10.1016/j.neuropsychologia.2012.02.028

[CR154] Vuust P, Heggli OA, Friston KJ, Kringelbach ML (2022) Music in the brain. Nat Rev Neurosci 23(5):287–305. 10.1038/s41583-022-00578-5 35352057 10.1038/s41583-022-00578-5

[CR158] Wang Y, Fernández-Miranda JC, Verstynen T, Pathak S, Schneider W, Yeh FC (2013) Rethinking the role of the middle longitudinal fascicle in language and auditory pathways. Cereb Cortex 23(10):2347–2356. 10.1093/cercor/bhs225 22875865 10.1093/cercor/bhs225

[CR159] Warren JE, Wise RJS, Warren JD (2005) Sounds do-able: auditory-motor transformations and the posterior temporal plane. Trends Neurosci 28(12):636–643. 10.1016/j.tins.2005.09.010 16216346 10.1016/j.tins.2005.09.010

[CR160] Wasserthal J, Neher P, Maier-Hein KH (2018) TractSeg - fast and accurate white matter tract segmentation. NeuroImage 183(March):239–253. 10.1016/j.neuroimage.2018.07.070 30086412 10.1016/j.neuroimage.2018.07.070

[CR161] Wiener M, Turkeltaub P, Coslett HB (2010) The image of time: a voxel-wise meta-analysis. NeuroImage 49(2):1728–1740. 10.1016/j.neuroimage.2009.09.064 19800975 10.1016/j.neuroimage.2009.09.064

[CR162] Zald DH, Zatorre RJ (2011) On music and reward. In: Gottfried J (ed) The neurobiology of sensation and reward. Taylor & Francis

[CR163] Zatorre RJ, Belin P (2001) Spectral and temporal processing in human auditory cortex. Cereb Cortex 11(10):946–953. 10.1093/cercor/12.2.140 11549617 10.1093/cercor/11.10.946

[CR164] Zatorre RJ, Chen JL, Penhune VB (2007) When the brain plays music: auditory-motor interactions in music perception and production. Nat Rev Neurosci 8(7):547–558. 10.1038/nrn2152 17585307 10.1038/nrn2152

[CR165] Zhang Y, Chen G, Wen H, Lu KH, Liu Z (2017) Musical imagery involves Wernicke’s area in bilateral and anti-correlated network interactions in musicians. Sci Rep 7(1):1–13. 10.1038/s41598-017-17178-4 29213104 10.1038/s41598-017-17178-4 PMC5719057

