# Supplementary material for: Music reward sensitivity is associated with greater information transfer capacity within dorsal and motor white matter networks in musicians
Source: Brain Struct Funct. 2024 Jul 25;229(9):2299–313. doi: 10.1007/s00429-024-02836-x (PMC11611946; doi:10.1007/s00429-024-02836-x)
Supplement: Supplementary file 1 — Supplementary Material 1 [file 429_2024_2836_MOESM1_ESM.docx]

**Supplementary Information**

**
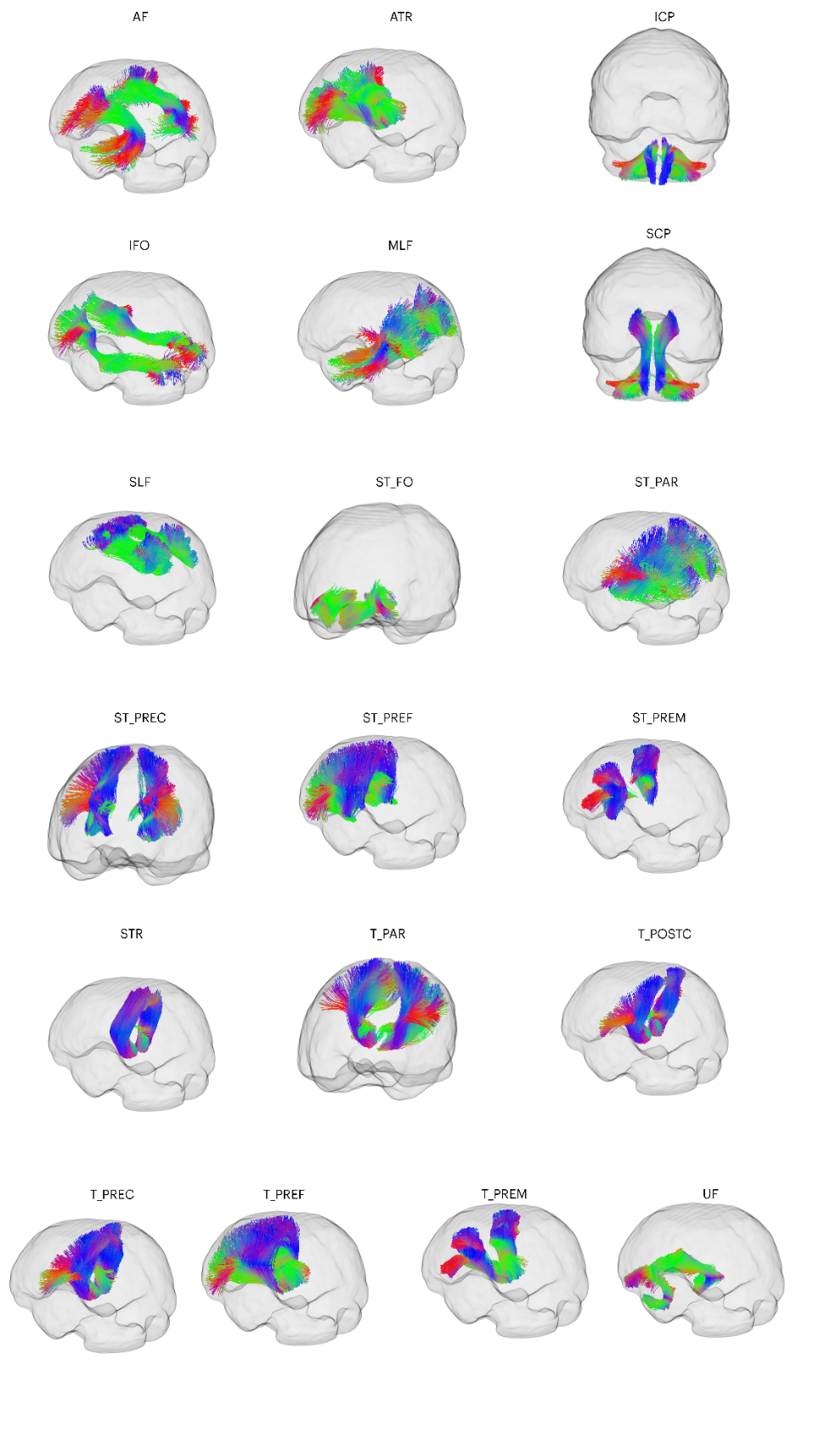
**

**Fig. S1** All tracts that were tested for a relation with MRS. These tracts were reconstructed by applying TractSeg to the study-specific population template, which had been transformed into MNI space using FA-based affine registration in FSL. Therefore, these images represent group-level data specific to the study sample. The images were created using MRtrix3. Note that SLF I, II, and III are combined in one plot. AF, Arcuate fasciculus; ATR, Anterior thalamic radiation; ICP, Inferior cerebellar peduncle; IFO, Inferior occipito-frontal fascicle; MLF, Middle longitudinal fascicle; SCP, Superior cerebellar peduncle; SLF, Superior longitudinal fascicle; ST_FO, Striato-fronto-orbital; ST_PAR, Striato-parietal; ST_PREC, Striatal-precentral; ST_PREF, Striato-prefrontal; ST_PREM, Striato-premotor; STR, Superior thalamic radiation; T_PAR, Thalamo-parietal; T_POSTC, Thalamo-postcentral; T_PREC, Thalamo-precentral; T_PREF, Thalamo-prefrontal; T_PREM, Thalamo-premotor; UF, Uncinate fascicle


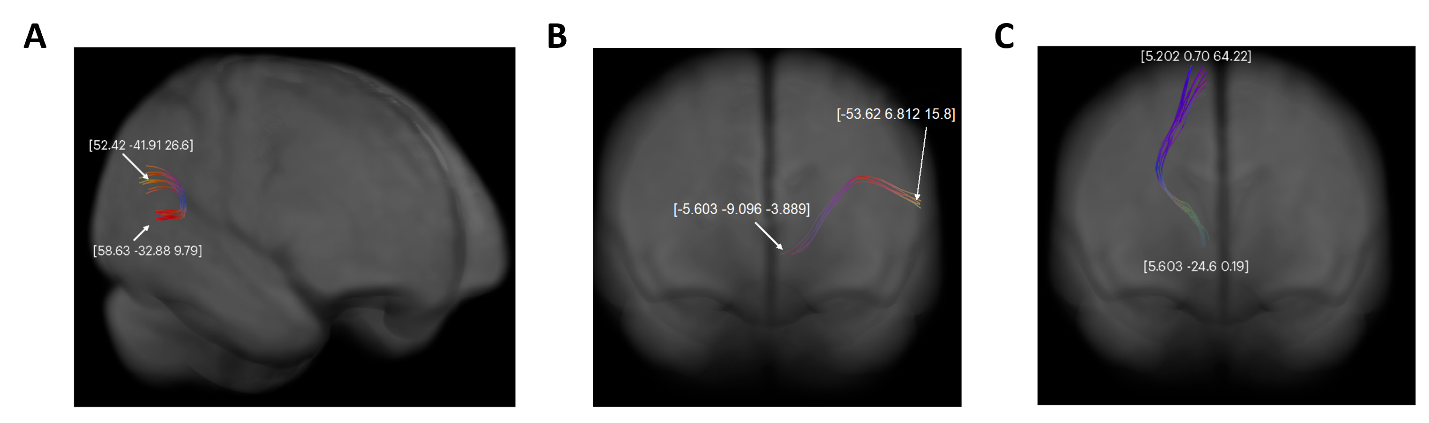


**Fig. S2** Tracts passing through significant fixels within A) the right middle longitudinal fascicle, B) the left thalamo-precentral, and C) the right thalamo-prefrontal tracts. Coordinates indicate estimated endpoints in MNI space.


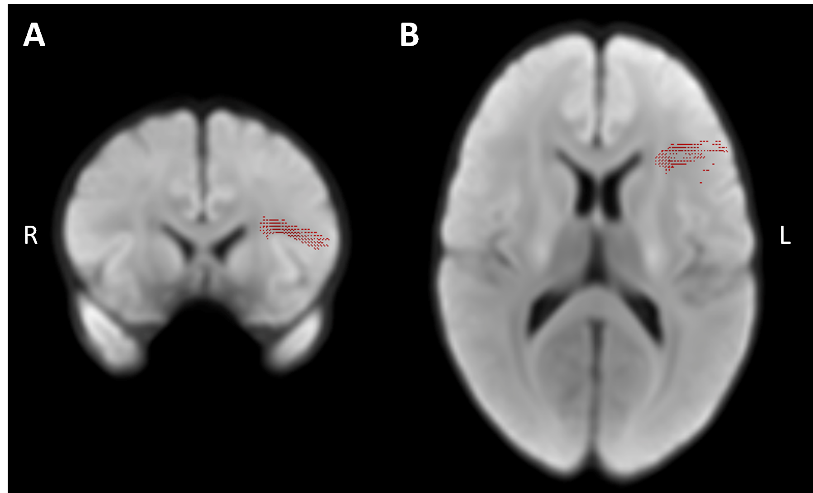


**Fig. S3** A) and B) Fixels within the left striato-thalamo tract for which FC showed a significant positive relation with years playing music.
